# Molecular basis of C-S bond cleavage in the glycyl radical enzyme isethionate sulfite-lyase

**DOI:** 10.1016/j.chembiol.2021.03.001

**Published:** 2021-09-16

**Authors:** Christopher D. Dawson, Stephania M. Irwin, Lindsey R.F. Backman, Chip Le, Jennifer X. Wang, Vyshnavi Vennelakanti, Zhongyue Yang, Heather J. Kulik, Catherine L. Drennan, Emily P. Balskus

**Affiliations:** 1 Department of Biology, Massachusetts Institute of Technology, Cambridge, MA 02139, USA; 2 Department of Chemistry and Chemical Biology, Harvard University, 12 Oxford Street, Cambridge, MA 02138, USA; 3 Department of Chemistry, Massachusetts Institute of Technology, Cambridge, MA 02139, USA; 4 Harvard Center for Mass Spectrometry, Faculty of Arts and Sciences Division of Science, Harvard University, 52 Oxford Street, Cambridge, MA 02138, USA; 5 Department of Chemical Engineering, Massachusetts Institute of Technology, Cambridge, MA 02139, USA; 6 Howard Hughes Medical Institute, Massachusetts Institute of Technology, Cambridge, MA 02139, USA; 7 Broad Institute, Cambridge, MA 02139, USA

**Keywords:** carbon-sulfur bond cleavage, glycyl radical enzyme, isethionate, sulfite, hydrogen sulfide, human gut microbiome, sulfate-reducing bacteria, *Bilophila wadsworthia*, lyase, structural enzymology

## Abstract

Desulfonation of isethionate by the bacterial glycyl radical enzyme (GRE) isethionate sulfite-lyase (IslA) generates sulfite, a substrate for respiration that in turn produces the disease-associated metabolite hydrogen sulfide. Here, we present a 2.7 Å resolution X-ray structure of wild-type IslA from *Bilophila wadsworthia* with isethionate bound. In comparison with other GREs, alternate positioning of the active site β strands allows for distinct residue positions to contribute to substrate binding. These structural differences, combined with sequence variations, create a highly tailored active site for the binding of the negatively charged isethionate substrate. Through the kinetic analysis of 14 IslA variants and computational analyses, we probe the mechanism by which radical chemistry is used for C-S bond cleavage. This work further elucidates the structural basis of chemistry within the GRE superfamily and will inform structure-based inhibitor design of IsIA and thus of microbial hydrogen sulfide production.

## Highlights

• Molecular basis for sulfite production in the human gut
• Crystallographic snapshots of a glycyl radical enzyme found in the human gut
• Molecular mechanism of radical-based C-S bond cleavage investigated
• Canonical glycyl radical enzyme β barrel is tailored to enable sulfonate binding

## Graphical abstract

**Figure:**
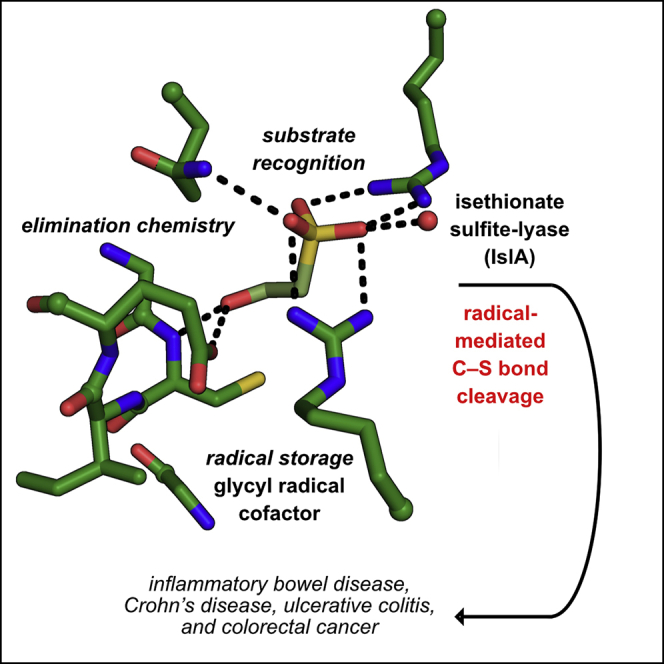

## Teaser

Dawson, Irwin et al. provide a molecular depiction of how sulfite, a precursor to the cancer- and Crohn's disease-associated metabolite hydrogen sulfide, is generated from isethionate by the enzyme isethionate sulfite-lyase in the human gut microbiota.

## Introduction

Certain gut bacteria release hydrogen sulfide as a byproduct of their respiration, which has implications for human health. Increased levels of hydrogen sulfide-producing bacteria are linked to a thinner colonic mucus barrier and multiple diseases, including inflammatory bowel disease (Ijssennagger et al., 2016), Crohn's disease, ulcerative colitis (Carbonero et al., 2012; Singh and Lin, 2015), and colorectal cancer (Yazici et al., 2017). Hydrogen sulfide levels in the human body depend largely on the gut microbiome (Shen et al., 2013) and have been implicated in circulatory system homeostasis (Tomasova et al., 2016) and antibiotic neutralization (Shatalin et al., 2011). One prominent bacterial species that generates hydrogen sulfide from sulfite is *Bilophila wadsworthia*. Isolated from fecal and appendicitis specimens and named for its ability to readily digest bile (Baron et al., 1989), *B. wadsworthia* is an opportunistic pathogen (Feng et al., 2017) and the third most common anaerobic bacterium isolated from removed appendices (Baron et al., 1992). Targeting hydrogen sulfide production by *B. wadsworthia* and other gut bacteria, such as sulfate-reducing bacteria (SRB), could become a therapeutic strategy to address these medical issues.

**Figure 1:**
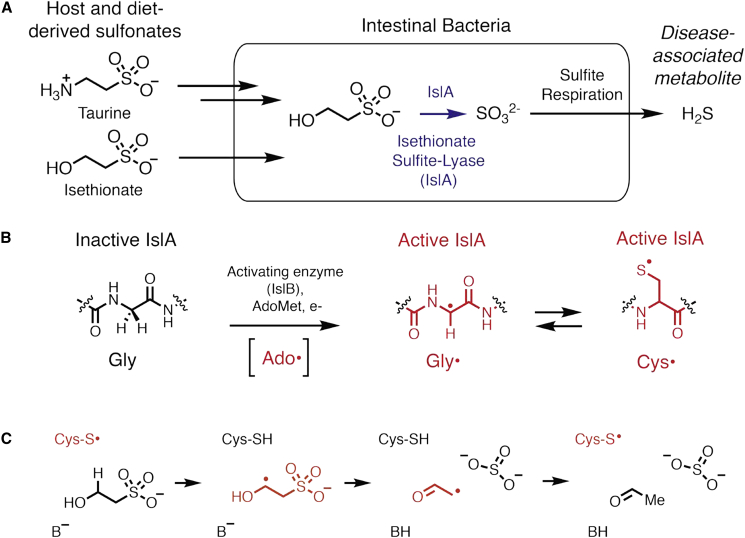
IslA-mediated anaerobic metabolism of organosulfonates by intestinal bacteria releases the disease-associated metabolite hydrogen sulfide (H_2_S) (A) Deamination of taurine by human gut microbes yields isethionate (Ise), which is cleaved and reduced to H_2_S in microbial respiration. (B) The activating enzyme for IslA, IslB, installs a glycyl radical on a particular glycine residue of IslA using radical SAM chemistry, i.e., the formation of a 5′-deoxyadenosyl radical (Ado⋅) species from the reductive cleavage of *S*-adenosylmethionine (AdoMet) using an one electron reduced [4Fe-4S] cluster. The glycyl radical (Gly⋅) transiently forms the catalytically essential thiyl radical species (Cys⋅). (C) Proposed reaction scheme for IslA. Radical species are shown in red.

To understand hydrogen sulfide production by *B. wadsworthia* we must understand the source of sulfur (Figure 1A). One critical source of sulfur is isethionate (2-hydroxyethanesulfonate, Ise), which is derived primarily from microbiome-dependent deamination of taurine, an abundant osmolyte in mammals and the second most abundant free amino acid in the human ileum and proximal colon (Smith and Macfarlane, 1998), as well as a conjugate of bile salts (Fellman et al., 1980). Recently, the enzyme responsible for catalyzing C-S bond cleavage of Ise to form acetaldehyde and sulfite, isethionate sulfite-lyase (IslA), was identified and biochemically characterized from *B. wadsworthia* (Peck et al., 2019), although isethionate lyase activity has been known for decades (Kertesz, 2000; Laue et al., 1997; Lie et al., 1996, 1999). IslA homologs are also found in the genomes of SRB from the human gut microbiome and other environments (Goldstein et al., 2003; Peck et al., 2019).

IslA belongs to the glycyl radical enzyme (GRE) superfamily, which performs diverse chemical reactions under anaerobic conditions (Backman et al., 2017). All characterized GREs share a ten-stranded α/β barrel architecture housing the active site, including two loops named for catalytically essential residues: the Cys loop and the Gly loop. Each GRE is activated by a dedicated radical S-adenosylmethionine (AdoMet)-dependent [4Fe-4S] activase (IslB for the IslA GRE) that installs a radical on a glycine residue on the Gly loop. During the reaction cycle this glycyl radical is thought to abstract a hydrogen atom from a conserved cysteine residue on the Cys loop, forming a catalytic thiyl radical (Figure 1B). This thiyl radical abstracts a hydrogen atom from the substrate to form a substrate radical that rearranges, forming a product radical. The radical is then transferred back to the catalytic Cys and subsequently to Gly, allowing for multiple rounds of turnover.

IsIA falls into the eliminase class of GREs (Peck et al., 2019), which also includes propane-1,2-diol dehydratase (LaMattina et al., 2016), B_12_-independent glycerol dehydratase (GD) (O'Brien et al., 2004), *trans*-4-hydroxy-L-proline (Hyp) dehydratase (HypD) (Levin et al., 2017), and choline trimethylamine-lyase (CutC) (Craciun and Balskus, 2012). By analogy to these other GRE eliminases, we hypothesize that IslA performs C-S bond cleavage on Ise through a 1,2-elimination mechanism (Figure 1C). Furthermore, CutC presents an interesting case for comparison with IslA, since their substrates are both functionalized ethanol derivatives (Figure S1). Both substrates also possess charged leaving groups, which present unique chemical challenges for their respective enzymes in terms of both substrate recognition and leaving group stabilization.

Recently, a crystal structure of IslA from *Desulfovibrio vulgaris* Hildenborough (DvIslA) was solved using a 23-amino acid N-terminal truncation and “surface-entropy reduction mutations” with residues 133–136 substituted with alanine residues (Xing et al., 2019), providing an initial view of an IsIA. Here, we present a full structure/function analysis of IsIA, in which we report the native IsIA structure, the Ise-bound structure of IsIA from *B. wadsworthia* 3.1.6 (IslA) at 2.70 Å resolution, along with the biochemical characterization of 14 enzyme variants. Collectively, these studies provide insight into how this enzyme performs C-S bond cleavage and into how substrate and reaction specificity are modulated in the GRE superfamily.

## Results

### Overall architecture of IslA is consistent with other GRE eliminases

**Figure 2:**
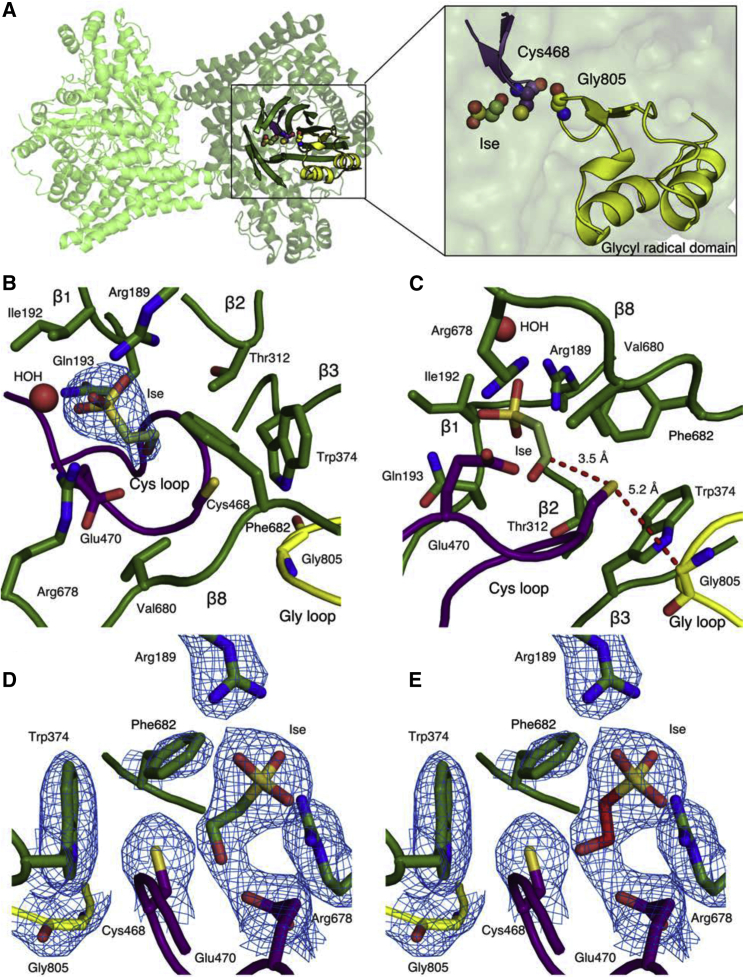
Overall architecture of *Bilophila wadsworthia* isethionate sulfite-lyase (IslA) (A) IslA dimer contains an active site comprised of two five-stranded half barrels enclosing substrate, the Cys loop (purple) and the Gly loop (yellow). Shown in spheres are Ise, and the catalytic cysteine and glycine residues. (B) Active site views of substrate-interacting residues with F_o_ − F_c_ composite omit map contoured to 1.5σ around Ise. (C) Active site view with proposed H atom abstraction route shown in red. (D) A different orientation of the Ise-bound IsIA structure and maps shown in (B). In this refined orientation of Ise (green carbons), it is the *pro*-R hydrogen on C2 that points toward Cys468. (E) When Ise (red carbons) is modeled into the map shown in (B) such that the *pro*-S hydrogen on C2 is pointing toward Cys468, the fit to the density is less good. (D) and (E) also show composite omit map contoured to 1.5σ (blue) for the side chains of residues that surround the substrate. Chain A was used to generate (D and E). Chain D was used for all other structure figures.

A structure of IslA from *B. wadsworthia* 3.1.6 was solved to 2.26 Å resolution by molecular replacement using CutC (PDB: 5FAU; Bodea et al., 2016) as the search model (Table 1) with 1.84 Å root-mean-square deviation (RMSD) to the CutC structure and 0.57 Å RMSD to the recently published DvIslA structure (PDB: 5YMR; Xing et al., 2019). During model refinement, positive difference density was observed in the active site that resembled glycerol, a component of the purification buffer and cryoprotectant (Figure S2A). After dialysis of the purified protein and increasing the isethionate concentration, a second IslA structure with the substrate Ise bound was obtained (Figures S2B–S2D). This structure was solved to 2.70 Å resolution by molecular replacement with the glycerol-bound IslA structure as the search model (Figure 2; Table 1).

**Table 1:** Crystallographic data and refinement statistics

Data name	Glycerol-bound IslA	Ise-bound IslA
**Data collection**		

Wavelength (Å)	0.9792	0.9792
Space group	P2_1_2_1_2	P2_1_2_1_2_1_
Cell dimensions		
a, b, c (Å)	119.99, 132.86, 107.72	130.95, 163.75, 181.65
Resolution (Å)	50–2.26 (2.34–2.26)^a^	50–2.70 (2.80–2.70)
No. of unique reflections	80,740 (6,838)	107,420 (10,625)
R_sym_ (%)	17.3 (49.5)	22.6 (102.4)
I/σ(I)	10.1 (2.0)	7 (1.4)
Completeness (%)	98.4 (84.8)	98.9 (99.2)
Redundancy	7.3 (3.2)	4.1 (3.8)
CC½	98.9 (68.1)	96.9 (49.6)

**Refinement**		

Resolution (Å)	50–2.26	50–2.70
No. of unique reflections	80,669	107,297
R_work_/R_free_^b^	0.166/0.198	0.184/0.223
No. of atoms	13,773	27,303
Protein	13,174	26,332
Glycerol	12	
Isethionate		28
Water	587	943
B factors (Å^2^) (overall)	26.0	36.7
Protein	26.4	36.8
Glycerol	23.2	
Isethionate		31.5
Water	27.0	34.5
RMSD		
Bond lengths (Å)	0.003	0.004
Bond angles (°)	0.589	0.623
Rotamer outliers (%)	1.2	0.94

RMSD, root-mean-square deviation.

a Highest-resolution shell shown in parentheses.

b R_free_ was calculated with 5% of the data.

As is the case for the core architecture of all characterized GRE eliminases (Backman et al., 2017), IslA is dimeric with each monomer having a buried active site located centrally within a barrel comprised of two five-stranded half β barrels, anti-parallel to each other (β1–10), and surrounded by α helices (Figure 2A). This buried active site is believed to shield radical species from solvent quenching (Backman et al., 2017). In the active site are two nearby and catalytically essential loops: the Cys loop and the Gly loop. All GREs have a C-terminal glycyl radical domain containing the Gly loop and its conserved Gly residue (Gly805 in IslA). The Cys loop includes the conserved catalytic Cys residue (Cys468 in IslA). Gly805 and Cys468 are 5.2 Å from each other (Figure 2C), competent for radical transfer from Gly805 to Cys468, and for the generation of a transient thiyl radical that initiates catalysis on the substrate.

### IslA active site is tailored to bind the negatively charged substrate Ise

**Figure 3:**
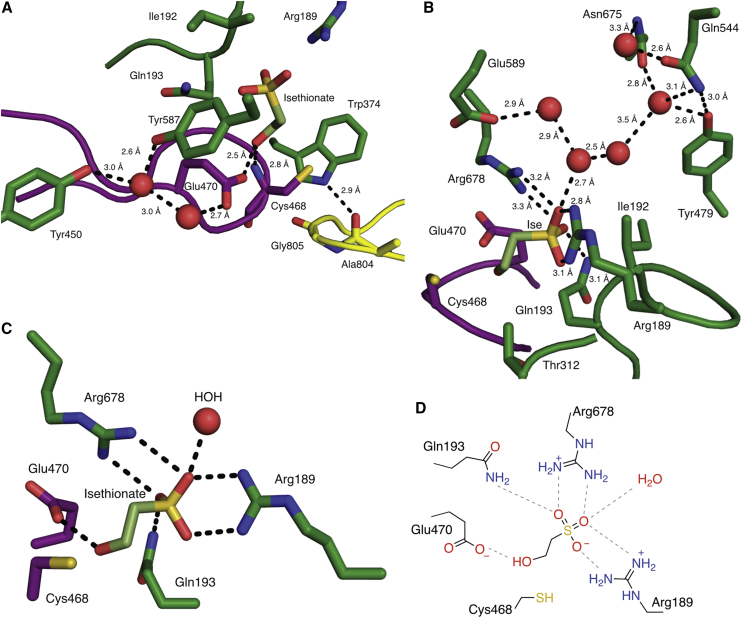
Ise binding mode in IslA (A) Residues and water molecules interacting with Ise hydroxyl group and Gly loop shown as dotted lines and waters shown as red spheres. (B) Residues and water molecules interacting with Ise sulfonate group. (C) Simplified active site view. (D) Active site hydrogen bond interaction scheme.

In the active site of the second IslA structure, electron density was observed for the substrate Ise (Figures 2B and S2). A Glu residue (Glu470) hydrogen bonds with the hydroxyl group of Ise with an additional hydrogen bond being provided by the amide of Cys468 (Figure 3A). Although the overall organization of IslA’s active site approximates those of other GRE eliminases, unique features enable Ise specific binding. Several polar and electrostatic residues stabilize the negatively charged sulfonate group of Ise: Arg189 and Gln193 of β1, as well as Arg678 of β8 (Figures 3B, 2D, and 2E), all of which are conserved in IslA homologs but not in other GREs according to sequence alignments performed using Clustal Omega (Sievers et al., 2011) (Figure S3). A water molecule that is present in all four molecules of the asymmetric unit provides another hydrogen bonding partner to the sulfonate group (Figure 3B). This water molecule itself is stabilized by a solvent pocket located peripherally to the sulfonate group. Overall, hydrogen bonding and electrostatic interactions form a unique active site to accommodate a highly charged, hydroxyl-containing molecule, such as Ise (Figures 3C and 3D).

### Ise positioning in IslA is unique among GRE eliminases

**Figure 4:**
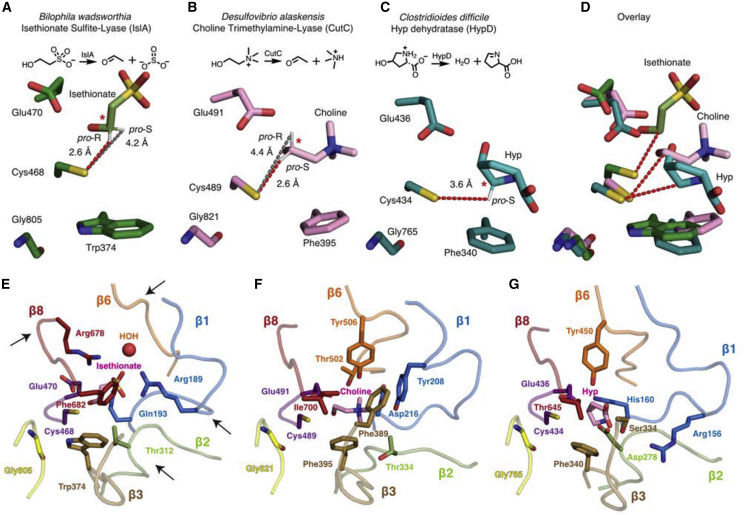
Substrate positioning and active site architecture differs among GRE eliminases (A) IslA shown in green with proposed radical transfer pathway shown as a red dotted line from the catalytic Cys (Cys468) to the closest substrate carbon marked with a red star. The gray dotted line indicates the distance between the more distant hydrogen of the substrate carbon. (B) CutC (PDB: 5FAU) shown in pink. (C) HypD (PDB: 6VXE) shown in teal. (D) Overlay of (A–C). (E) IslA with substrate, Gly loop, Cys loop, β1, β2, β3, β6, and β8 shown as pink, yellow, purple, blue, green, brown, orange, and red, respectively. Arrows highlight particularly different β strands. (F) CutC (PDB: 5FAU) colored as in (E). (G) HypD (PDB: 6VXE) colored as in (E).

A conserved feature of GRE eliminases is the CXE motif of the Cys loop (Cys468-Ile469-Glu470 in IslA) (Figures 2B and 2C). Here, as in all previous GRE eliminase structures (Backman et al., 2017), the substrate sits above the Cys loop (Figure 2C) and the Glu residue of the CXE motif appears to hydrogen bond with a hydroxyl group of substrate (Figures 3A and 3C). However, in comparison with CutC (Bodea et al., 2016) and HypD (Backman et al., 2020), the position of Ise is shifted, and the orientation of the hydroxyl of Ise relative to the carboxylate of Glu470 is also shifted to accommodate the unique position of Ise (Figures 4A–4D). If the orientation of Glu470 mimicked that of the Glu residues in other GRE eliminases, it would crash into the Ise substrate (Figure 4D). Even though Ise and choline are more structurally similar to each other than either is to Hyp, it is the active site of IsIA that is the outlier. Another difference generated by the unique Ise positioning is that the *pro*-*R* hydrogen of C2, rather than the *pro*-*S* hydrogen, is closer to the thiol of Cys468, with distances of 2.6 and 4.2 Å, respectively (Figures 4A and 2D). Attempts to re-position Ise such that the *pro-S* hydrogen of C2 is closer to Cys468 result in a poor fit to the electron density (Figure 2E). In contrast, in most other structurally characterized GRE eliminases, the thiol of the catalytic cysteine residue is closer to the *pro*-*S* hydrogen atom.

Another common feature of GRE active sites is that an aromatic residue packs against the substrate (Backman et al., 2017). In CutC, Phe395 provides cation-π interactions to the positively charged choline, and similarly in HypD, Phe340 is positioned to make cation-π interactions with the amino group of Hyp (Figures 4B and 4C). Interestingly, an unexpected feature of IslA is the presence of a Trp residue (Trp374) in the position typically occupied by Phe. Trp374 is too far (∼6 Å) to directly interact with substrate but, due to its larger size, it does sit closer to Gly805 and Cys468 than is common (Figures 4A–4D). Although an aromatic residue is typically found in this position, only IsIA enzymes have Trp among GRE eliminases and, within the putative IsIA enzyme family, Trp374 is conserved (Figure S3). Although the functional role of Trp in IsIA is unknown, the presence of Trp versus Phe does allow for an additional hydrogen bond between the Trp side chain and the glycyl radical loop (Figure 3A), which could regulate the movement of the glycyl radical loop out of the active site for glycyl radical formation.

### Alterations in barrel architecture modulate substrate specificity

In GREs, substrate-binding residues are introduced into the active site by the β strands and, when the strands adopt even subtly different orientations, the effect on the active site can be substantial (Figures 4E–4G). Specificity in GREs is thus determined both by the substitution of residues on similarly positioned parts of β strands and by the active site alterations created by β strands repositioning. The biggest rearrangement of a β strand in IslA is found in β6, which allows Ise to bind in a much higher up position in the active site than those of Hyp or choline in their respective enzymes (see above). In CutC (Bodea et al., 2016) and HypD (Backman et al., 2020), β6 runs closer to the active site, positioning Tyr506 and Tyr450, respectively, toward the substrate (Figures 4F and 4G). In IsIA, β6 departs the active site more abruptly, creating a water-binding site and room for substrate Ise to sit higher in the active site (Figure 4E). To secure Ise in this “higher” substrate-binding position, Arg678 extends into the active site from a position on β8 too distant to interact with substrate in CutC and HypD. Arg678 appears to be important in making a favorable electrostatic interaction with the substrate sulfonate group. β8 of IslA also contributes Phe682, which occupies a similar space as a β3 residue in CutC (Phe389), fulfilling a van der Waals packing role with the same type of residue from a different strand. In contrast, in HypD the corresponding residues to Phe682-IsIA and Phe389-CutC are Thr645 and Ser334, both hydrogen bond donors to substrate Hyp.

Strands β1 and 2 also contribute residues to the active sites of all three of these GRE enzymes (Figures 4E–4G). Like β6, the conformation of β2 can be quite different in different GRE eliminases (Figures 4E–4G). Also, the importance of β2 residues to substrate binding and/or catalysis can differ considerably. An essential catalytic residue in HypD is contributed by β2 (Asp278) (Backman et al., 2020), whereas IsIA-Thr312 and CutC-Thr334 do not directly contact substrate (Figures 4E–4G). In CutC, a different (lower) conformation of β2 positions Thr334 too far from substrate, whereas in IsIA, a different (higher) position of substrate puts Thr312 out of reach (Figures 4E–4G). It is clear from comparing structures of eliminases that the flexibility of GRE active sites is quite substantial.

Finally, β1 residues appear to be key players in all three enzymes. A common site is used that contributes substrate-interacting residues: IslA-Gln193, CutC-Asp216, and HypD-His160 (Figures 4E–4G). In contrast, the site on β1 of CutC that provides substrate-binding residue Tyr208 is not used by either HypD or IslA, but both HypD and IslA use an upstream site to contribute an Arg residue. Although this Arg (IsIA-Arg189 and HypD-Arg156) is from the same position on the backbone, the side-chain orientations are quite different. IslA-Arg189 swings to interact directly with Ise, whereas HypD-Arg156 interacts indirectly with Hyp through a water molecule. Overall, comparing IslA, CutC (Bodea et al., 2016), and HypD (Backman et al., 2020) showcases the extraordinary ability of GREs to tailor interactions with different substrates using the same GRE β barrel architecture.

### A similar putative substrate channel is found in several GRE structures

The active sites of GREs are relatively buried, which serves to protect the radical species from oxygen damage. Due to the buried nature of the active site, substrate access channels and product release channels are required. The clearest example of a substrate channel is found in the GRE benzylsuccinate synthase (BSS) (Funk et al., 2014, 2015), which must accommodate entry of a volatile and hydrophobic aromatic compound, toluene, as well as a polar molecule, fumarate. BSS has accessory subunits that are required in addition to the catalytic α subunit, a feature shared with another characterized GRE, 4-hydroxyphenylacetate decarboxylase (HPAD) (PDB: 2Y8N; Martins et al., 2011). In the BSS-αβγ structure (PDB: 4PKF; Funk et al., 2014, 2015), a hairpin loop of a non-catalytic subunit (BSS-β) plugs the putative substrate access channel in the catalytic subunit (BSS-α), closing off the active site once substrate is bound (Figure S4A,B). Thus, the structures of the open BSS-αγ and closed BSS-αβγ enable us to visualize substrate channel closure in a GRE.

**Figure 5:**
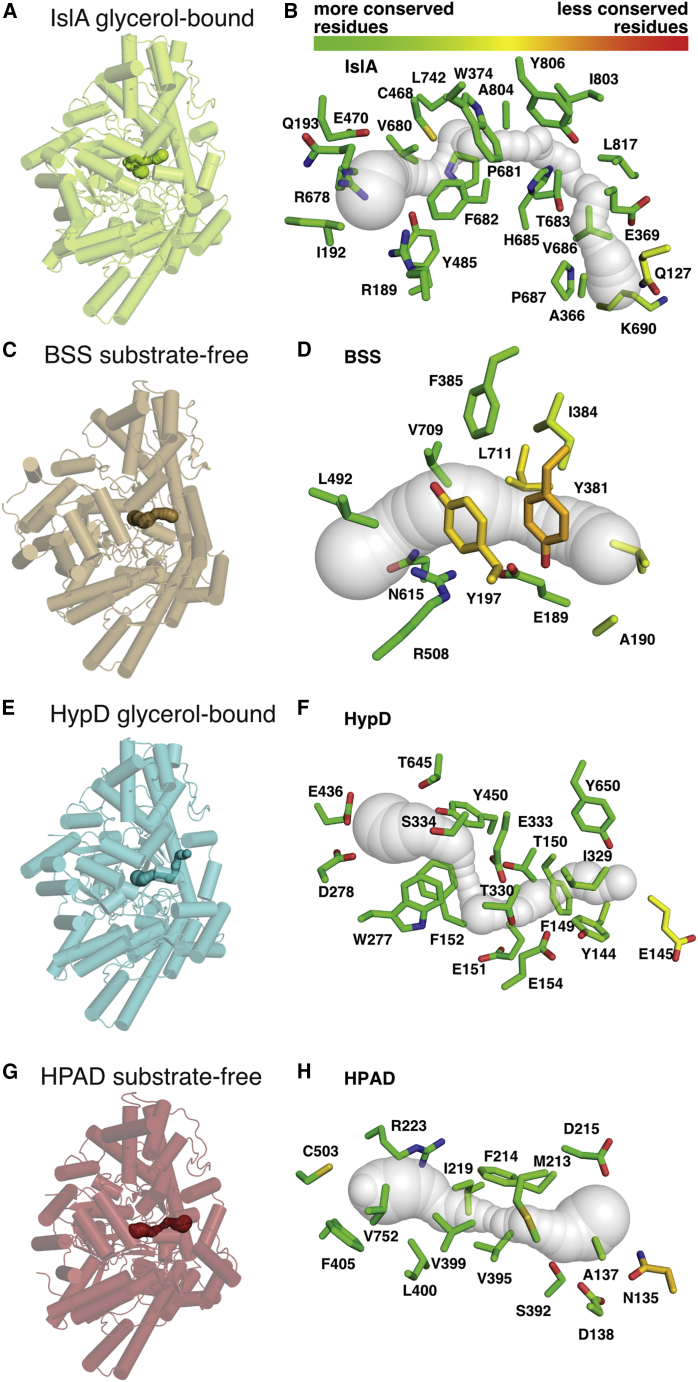
Several putative GRE substrate channels share a similar location (A) One monomer of the IslA dimer is shown in green and oriented similarly to other GRE enzyme monomers, which are shown transparent with channels identified using CAVER 3.0, shown opaque. (B) Conservation of residues of substrate channel (transparent surface) of glycerol-bound IslA. Residues within 4 Å of the putative substrate channel are shown as sticks and colored by conservation score as computed by ConSurf (Ashkenazy et al., 2016) with a gradient from lower scores (more conserved) show in green to higher scores (less conserved) shown in red. (C) BSS (PDB: 4PKC) α subunit (brown). (D) Same representation as (B), but for BSS α subunit (PDB: 4PKC). (E) HypD (PDB: 6VXE) (teal). (F) Same representation as (B), but for HypD (PDB: 6VXE). (G) HPAD (PDB: 2Y8N) α subunit (red). (H) Same representation as (B), but for HPAD α subunit (PDB: 2Y8N).

To investigate whether other GREs have a similar channel to that of BSS, we ran an analysis of available GRE structures using the program CAVER 3.0 (Chovancova et al., 2012) and compared channels identified with the toluene channel of BSS (Figure 5). We find an equivalent channel to that in BSS in the two glycerol-bound structures of HypD and IslA (Figure 5). Furthermore, this putative channel is also seen in the substrate-free structure of HPAD-αγ (Martins et al., 2011). The lengths of these channels range between 14 and 19 Å from the active site to the protein surface. Residues along the channels are highly conserved in each of these four GREs, consistent with these channels playing a functional role (Figure 5). Considering the many architectural differences among these GREs, a consistent channel is an interesting feature worthy of further experimental validation.

### Site-directed mutagenesis experiments validate Ise-coordinating residues as playing roles in substrate binding and catalysis

Using our structural data, we sought to probe the roles of active site residues through site-directed mutagenesis experiments. We validated that Gly805 forms the glycyl radical and that Cys468 is catalytically essential by generating G805A and C468S variants of IslA (Figures S5A and S5B). Unsurprisingly, electron paramagnetic resonance (EPR) spectroscopy of the G805A variant detects no glycyl radical species (Table 2; Figure S6). The C468S variant can form a glycyl radical but has no detectable sulfite release in an endpoint assay (Figures S5C and S5D), as expected considering its predicted role in catalysis.

**Table 2:** Activation and kinetics of IslA variants from reactions repeated in triplicate

Protein	Percentage of active sites containing glycyl radical (%)	Detectable activity (sulfite)	K_M_ (mM)	k_cat_ (s^−1^)	Glycyl radical normalized k_cat_ (s^−1^)	Catalytic efficiency using normalized k_cat_ (s^−1^ M^−1^)
Wild-type	20.8 ± 0.5	yes	8 ± 2	2.0 ± 0.1	9.5 ± 0.6	1200 ± 310
G805A	0	no	N/D	N/D	N/D	
C468S	22.1 ± 0.4	no	N/D	N/D	N/D	
E470Q	11.2 ± 0.7	no	N/D	N/D	N/D	
Q193A	17 ± 1	no	N/D	N/D	N/D	
R189E	28.1 ± 0.6	no	N/D	N/D	N/D	
R678E	5.9 ± 0.1	no	N/D	N/D	N/D	
R189E/R678E	11 ± 1	no	N/D	N/D	N/D	
F682A	11 ± 1	yes	6.8 ± 0.6	0.0171 ± 0.0004	0.154 ± 0.004	23 ± 2.1
F682Y	32.2 ± 0.8	yes	8 ± 1	0.0074 ± 0.0003	0.023 ± 0.001	2.9 ± 0.4
W374F	47 ± 2	yes	11 ± 1	0.063 ± 0.003	0.133 ± 0.006	12 ± 1.2
W374Y	30.2 ± 0.9	yes	16.0 ± 0.9	0.140 ± 0.003	0.465 ± 0.009	29 ± 1.7
I192A	21 ± 1	yes	12 ± 2	0.40 ± 0.02	1.9 ± 0.1	160 ± 28
V680A	52 ± 3	no	N/D	N/D	N/D	

Percentage of active sites containing glycyl radical indicate mean ± standard deviations; kinetic parameters are listed as mean ± standard error. N/D, not determined

Next, we examined residues observed to interact with Ise in the crystal structure. We made variants of IslA to disrupt putative interactions with the hydroxyl group of Ise (E470Q) and the sulfonate moiety of Ise (Q193A, R189E, R678E, and R189E/R678E). All five enzyme variants were activated by IslB to some extent, with R189E having the greatest glycyl radical content (Table 2; Figure S6). However, none of the five variants displayed endpoint activity (Figures S5C and S5D).

We also investigated the aromatic residues in the active site (F682A, F682Y, W374F, and W374Y) (Figures 2B and 2C). IsIB successfully installed a glycyl radical into these enzyme variants as determined by EPR spectroscopy, and the endpoint assay indicated turnover (Table 2; Figure S5C, S5D, and S6). To determine the effects these mutations have on catalysis, kinetic assays were conducted for wild-type (WT) IslA and the IslA variants F682A, F682Y, W374F, and W374Y (Figure S7). The K_M_ value for the WT *B. wadsworthia* IslA (8 ± 2 mM) is higher than the K_M_ values from some GREs, such as CutC (0.13 mM) or HypD (1.2 mM) (Craciun et al., 2014; Levin et al., 2017). It is comparable with the other published IslA *K*_M_ values from *D. desulfuricans* DSM642 (6.3 mM) and lower than that for *D. vulgaris* (44.8 mM) (Peck et al., 2019; Xing et al., 2019) and lower than the K_M_ value from another recently discovered C-S bond-cleaving GRE, HpsG (13 mM) (Liu et al., 2020). Although the concentrations of Ise or precursor taurine have not been reported for the duodenal mucus or colonic mucus to the best of our knowledge, biopsy data indicate that the amount of taurine in the intestinal mucus is high (6.12 and 2.49 mmol/kg for duodenal mucus and colonic mucus, respectively) (Ahlman et al., 1993a, 1993b). If we assume a density of 1 kg/L, taurine concentrations of 6 and 2.5 mM are of the same order of magnitude as our measured K_M_ (8 ± 2 mM). In addition, *B. wadsworthia* strains have been shown to adhere to human intestinal cells *in vitro*, indicating that they have access to this taurine pool (Gerardo et al., 1998).

The catalytic efficiencies of each of the IslA variants F682A, F682Y, W374F and W374Y, were 1–2 orders of magnitude lower than WT IslA due largely to decreased k_cat_ with little difference in K_M_, suggesting that these amino acids play a larger role in catalysis than in substrate binding. Phe682 appears to be important for catalysis, perhaps through controlling the substrate conformation, although it is not necessary for Ise cleavage. Trp374 is closer to the catalytic cysteine (3.5 Å from the thiol of Cys468) and the glycyl radical (3.5 Å from the carbonyl of Gly805) than to substrate (∼6 Å). The side chain of Trp374 is within hydrogen bond distance of the carbonyl of Ala804 (Figure 3A), providing a direct interaction with the Gly loop. Interestingly, W374F and W374Y variants display more glycyl radical content than WT but are less active (Table 2; Figures S6 and S7). It could be that the loss of the hydrogen bond to the Gly loop through residue substitution increases the dynamics of the Gly loop, and the results of increased dynamics are 2-fold. Activation, which requires Gly loop movement, is increased, whereas radical transfer between Gly and Cys, which requires a close positioning of the Gly loop relative to Cys, is impaired.

Finally, we mutated the additional residues Ile192 and Val680. Ile192 is near the sulfonate group of the Ise substrate, whereas Val680 is located ∼5 Å from Ise but in close proximity (∼3 Å) to the catalytically important Cys468 (Figures 2B and 2C). I192A was found to have nearly identical radical installation to the WT, and a similar K_M_ with a decreased k_cat_ (Table 2; Figures S6 and S7). However, this mutant had the highest k_cat_ of the panel of mutants assayed, suggesting that Ile192 is less critical for catalysis. This finding is further supported by its lack of conservation among IslA homologs (Figure S3). Surprisingly, V680A was found to have the maximal amount of glycyl radical installation of the assayed mutants despite being inactive toward Ise (Figure S6). At 52% ± 3% radical installation per polypeptide, this variant has also reached what is believed to be maximal radical installation. In brief, it is thought that GREs use half-site reactivity, although this hypothesis requires additional experimental evidence (Backman et al., 2017). It is unclear if this increase in glycyl radical activation for this variant or others is due to altered interaction between the activase and IslA or to altered stability of the Gly radical. Further investigation is needed to determine the role of this residue, but this finding highlights how small structural changes relatively far from the substrate can dramatically impact catalysis.

### Deuterium-labeling studies show that the abstracted hydrogen atom is returned to the product

**Figure 6:**
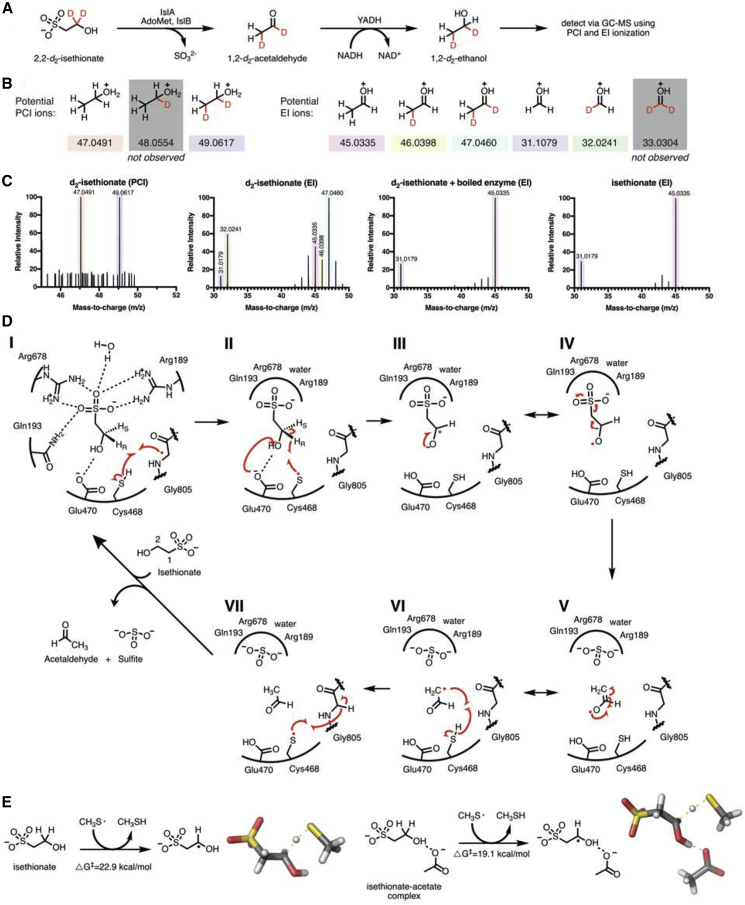
Biochemical and computational support for proposed IsIA mechanism (A) Schematic of IsIA GC-MS assay with stable-isotope-labeled Ise. (B) Potential positive chemical ionization (PCI) ions to determine total deuterium incorporation and potential electron impact (EI) ions used to identify the location of the deuterium atoms. (C) MS data from GC-MS assays. The enzymatic reaction with 2,2-d_2_-Ise generates 1,2-d_2_-ethanol as the only detectable deuterated product. Unlabeled ethanol (m/z = 47.0491) and d_2_-ethanol (m/z = 49.0617) are detected via PCI when IslA reacts with 2,2-d_2_-Ise. Using EI ionization and 2,2-d_2_-Ise as a substrate for IslA, the ion at 47.0460 represents di-deuterated ethanol. There is a +1 shift of the 31.0179 fragment ion of this product to 32.0241, indicating only a single deuterium is on the C1 fragment, as double-deuterated 33.0304 is not observed. We have located this deuterium to the C, rather than the O, since the O-H bond is generated via the yeast alcohol dehydrogenase-catalyzed reduction reaction that takes place in aqueous buffer. Thus, the 46.0398 ion arises from a loss of deuterium due to ionization and does not correspond to 1-d_1_-ethanol. The reaction of IslA with unlabeled isethionate generates unlabeled ethanol (m/z = 45.0335 and 31.0179). When boiled enzyme is mixed with d_2_-Ise, unlabeled ethanol is present in the background, likely due to our multi-user anaerobic chambers. These assays were performed in triplicate, and a representative spectrum is shown. (D) Proposed 1,2–elimination mechanism of IslA. During the catalytic cycle, the glycyl radical (Gly805) abstracts a hydrogen atom from Cys468, forming a thiyl radical (I, II). This thiyl radical abstracts the *pro-R* hydrogen atom from C2 of Ise. Glu470 deprotonates the hydroxyl group of Ise to form a transient substrate ketyl radical species (III, IV). This unstable intermediate decomposes, resulting in C-S bond cleavage and release of sulfite (V). The resulting radical species then abstracts a hydrogen atom from Cys468 to produce the second product, acetaldehyde (VI, VII). Arrows are shown in red. Hydrogen bonds are shown in dashed black. (E) Schematic of the local coupled cluster quantum mechanical calculations of C-H abstraction from Ise both with and without an acetate molecule present in a dielectric medium. Spatially resolved structures of the transition states are shown beside each schematic.

To better understand the mechanism of IslA, we performed deuterium-labeling studies to determine if the hydrogen atom abstracted from Ise is returned to the product or is lost to solvent quenching or exchange. We incubated activated IslA with either unlabeled or 2,2-d_2_-Ise in a coupled assay with yeast alcohol dehydrogenase to generate ethanol as a final product (Figure 6A). Gas chromatography-mass spectrometry (GC-MS) analysis using positive chemical ionization revealed the formation of di-deuterated ethanol (Figures 6B and 6C). GC-MS with electron impact (EI) ionization was used to assign this product as 1,2-d_2_-ethanol (Figures 6B and 6C). These observations are consistent with the deuterium abstracted from 2,2-d_2_-Ise returning to the product during catalysis. The absence of a singly deuterated ethanol product also indicates a lack of deuterium exchange with solvent.

## Discussion

The structure of WT IsIA from *B. wadsworthia* with Ise bound has allowed us to compare how this GRE binds substrate with how other GRE eliminases position their substrates for catalysis. We find that IslA positions substrate higher in the active site than is typical for GREs that perform similar heteroatom elimination reactions. This higher positioning enables interactions with Arg678 and Arg189, which serve to counter the negative charge of the Ise sulfonate group. Interestingly, the unique binding position of Ise is not due solely to the identity of residues in the active site; the positioning of β strands of the ten-stranded barrel is also different, allowing residues from atypical positions on these strands to contribute to the active site. The combination of the repositioning of β strands with residue substitutions adds to the malleability of the active sites of GRE enzymes such that a higher degree of tailoring is possible. This malleability also leads to difficulty in bioinformatically predicting the types of chemistry and substrates a GRE of unknown function might perform, highlighting the critical need for structural characterization.

We can also compare the binding mode of Ise with that of other sulfonate compounds. In the Protein DataBank, we find multiple sulfonate-containing compounds bound to proteins, including molecules derived from crystallization conditions that are adventitiously bound to proteins and enzyme inhibitors that are bound to their target enzymes (Figure S8A). Physiologically relevant bound sulfonates are restricted to the following metabolites: Ise, taurine, and sulfolactate (Figure S8B). A survey of sulfonate-binding modes suggests that common strategies include the use of water molecules, backbone amides, or arginine and asparagine residues to form hydrogen bonds and electrostatic interactions (DiDonato et al., 2006; Nishiyama et al., 2016; O'Brien et al., 2003; Rossocha et al., 2005; Xing et al., 2019; Zhou et al., 2019). Unique to IslA is a binding mode that provides a glutamine residue and two arginine residues to coordinate the sulfonate moiety. The way IslA binds Ise is not only distinct among GRE eliminases but also unique among sulfonate-binding proteins.

The radical-based cleavage of Ise would be expected to occur via either a direct elimination reaction or via a migration reaction. Based on biochemical and computational data for CutC and GD, these enzymes have been proposed to perform direct elimination chemistry (Bodea et al., 2016; Feliks and Ullmann, 2012; Kovačević et al., 2018; O'Brien et al., 2004; Yang et al., 2019). In contrast, the adenosylcobalamin-dependent ethanolamine ammonia-lyase (EAL) has been proposed to perform a radical-based migration reaction (Toraya, 2003). Notably, we showed previously that IslA does not turnover a substrate analog that would be expected to cleave via a migration rather than elimination reaction (Peck et al., 2019). For that substrate analog, 2-hydroxyethyl-phosphonate, the phosphonate moiety would be expected to migrate rather than be cleaved by elimination, based on work with hydroxylpropylphosphonate epoxidase (Chang et al., 2013). Although we have not ruled out that IsIA’s lack of activity on hydroxylethylphosphate is due to a lack of binding, inspection of the IsIA structure suggests that the active site of IsIA should bind hydroxylethylphophonate but that the active site is not designed to enable a migration reaction. In particular, comparing the placement of residues in the active sites of CutC, EAL, and IsIA shows that CutC and IsIA active sites contain residues that would appear to sterically prevent migration chemistry (Gln193 and Thr312 in IslA, and Thr502 in CutC), whereas EAL has a residue to facilitate migration (Glu287) (Figure S9) (Bodea et al., 2016; Mori et al., 2014; Shibata et al., 2010; Toraya, 2003).

Using the above considerations along with our biochemical data, we propose a mechanism for IslA that involves direct elimination of the sulfonate moiety of Ise to generate acetaldehyde and sulfite. After substrate binding, during the catalytic cycle, the glycyl radical abstracts a hydrogen atom from Cys468 forming a thiyl radical (Figure 6D, step I). Based on the fit to the electron density of Ise (Figures 2D and 2E) and distances from the thiol of Cys468 to hydrogen atoms on C2 of Ise, we predict thiyl radical hydrogen atom abstraction at the *pro-R* position (Figure 4A; Figure 6D, step II). This is in contrast to most other GRE eliminases, which are proposed to abstract the *pro-S* hydrogen atom of substrate based on structural, biochemical, and computational data (Backman et al., 2020; Bodea et al., 2016; Feliks and Ullmann, 2012; Kovačević et al., 2018; LaMattina et al., 2016; O'Brien et al., 2004; Yang et al., 2019). However, the structure of a recently characterized C-S-cleaving GRE HpsG also predicts abstraction of the corresponding stereochemically positioned hydrogen atom of its substrate, suggesting that this could be a common feature of this group of GREs (Liu et al., 2020).

Accompanying this hydrogen atom abstraction, we propose a deprotonation of the hydroxyl of Ise facilitated by Glu470 (Figure 6D, step II) as is suggested for CutC (Bodea et al., 2016) and as is consistent with complete loss of activity in the E470Q variant. Further supporting this proposal, high-level quantum mechanical calculations on substrate models with the enzyme environment approximated by dielectric screening show that the presence of an acetate molecule (representing the Glu470 side chain) stabilizes the transition state for C-H bond abstraction by 3.8 kcal/mol (Figure 6E; Tables S1–S3). We further propose that the unstable, transient ketyl radical species generated in this step decomposes, resulting in C-S bond cleavage and the generation of the sulfite product (Figure 6D, steps III, IV, and V). The resulting radical species rearranges and abstracts a hydrogen atom from Cys468 to regenerate the thiyl radical and to produce the acetaldehyde product, as supported by the deuterium-labeling experiments (Figure 6D, steps V and VI). The thiyl radical abstracts a hydrogen atom from glycine to reform the glycyl radical for the next round of catalysis (Figure 6D, step VII). The products, acetaldehyde and sulfite, are released, potentially through a highly conserved channel seen in IslA and other GREs (Figure 5), allowing for the next Ise substrate to bind. Thus, IslA uses radical-based chemistry to break a carbon-sulfur bond of a sulfonate group, a previously uncharacterized enzyme reactivity.

This structural and biochemical analysis of IslA has enabled us to identify residues important for substrate binding and catalysis and to explore how an opportunistic pathogen extracts sulfite from host-derived metabolites to fuel respiration. These data will aid in the identification of GREs as putative IslA enzymes and will enable drug design efforts toward reducing the negative effects of microbially derived hydrogen sulfide. Using IslA, microbes have devised a way to extract sulfite from a sulfonate common in biological systems, potentially to the detriment of their hosts.

## Significance

**An overabundance of hydrogen sulfide released during sulfite respiration by the gut microbiome is associated with diseases in the human host. The recently discovered glycyl radical enzyme isethionate sulfite-lyase (IslA) enables microbes to extract sulfite from isethionate, a derivative of the abundant metabolite taurine. Here, we identify residues important for binding and catalysis to expand our mechanistic understanding of IslA-mediated C-S cleavage toward the ultimate goal of structure-based inhibitor design of IsIA and thus of hydrogen sulfide production.**

## STAR★Methods

### Key resources table

**Table:** 

REAGENT or RESOURCE	SOURCE	IDENTIFIER
**Bacterial and virus strains**		

*E. coli* BL21(DE3) Δ*iscR*	Peck et al., 2019	N/A
*E. coli* BL21(DE3)	Invitrogen	Cat#C600003
*E. coli* TOP10	Invitrogen	Cat#C404003

**Biological samples**		

Chemicals, Peptides, and Recombinant Proteins		

EDTA-free protease inhibitor tablet	Sigma Aldrich	Cat#S8830
*N*-(9-acridinyl)maleimide	TCI America	Cat#A5591
Isopropyl β-D-1-thiogalactopyranoside (IPTG)	Teknova	Cat#I3325
*S*-(5-Adenosyl)-L-methionine p-toluenesulfonate salt	Sigma Aldrich	Cat#A2408
Isethionic acid sodium salt	Sigma Aldrich	Cat#220078
Acriflavine	Sigma Aldrich	Cat#A8251
Frémy salt K_2_(SO_3_)_2_NO	Sigma Aldrich	Cat#220930
Yeast Alcohol Dehydrogenase	Sigma Aldrich	Cat# A3263
β-Dihydronicotinamide adenine dinucleotide disodium salt (NADH-Na₂) trihydrate	VWR	Cat# 97061-534

**Critical commercial assays**		

E.Z.N.A. Plasmid Mini Kit I	Omega Bio-tek	Cat# D6943-02

**Deposited data**		

Glycerol-bound *B. wadsworthia* IslA structure	This Paper	PDB ID: 7KQ4
Isethionate-bound *B. wadsworthia* IslA structure	This Paper	PDB ID: 7KQ3
Choline-bound choline TMA-lyase (CutC) structure	Bodea et al., 2016	PDB ID: 5FAU
*D. vulgaris* IslA structure	Xing et al., 2019	PDB ID: 5YMR
Hydroxyphenylacetate decarboxylase (HPAD) structure	Martins et al., 2011	PDB ID: 2Y8N
Benzylsuccinate synthase (BSS) alpha-beta-gamma complex structure	Funk et al., 2014	PDB ID: 4PKF
BSS alpha-gamma complex structure	Funk et al., 2014	PDB ID: 4PKC
Hydroxyproline-bound hydroxyproline dehydratase (HypD) structure	Backman et al., 2020	PDB ID: 6VXE
Sulfoacetaldehyde reductase (KolsfD) structure	Zhou et al., 2019	PDB ID: 6IXJ
2-Phosphosulfolactate phosphatase (ComB) structure	DiDonato et al., 2006	PDB ID: 1VR0
Taurine:2OG dioxygenase (EcTauD) structure	O’Brien et al., 2003	PDB ID: 1OS7
Taurine chemoreceptor (VcMlp37) structure	Nishiyama et al., 2016	PDB ID: 5AVF
Bile acid hydrolase (CpCBAH) structure	Rossocha et al., 2005	PDB ID: 2BJF

**Oligonucleotides**		

See Table S4 for DNA Oligonucleotides	This Paper	N/A

**Recombinant DNA**		

pET-28a-IslA	Peck et al., 2019	N/A
pET-29b-IslB	Peck et al., 2019	N/A
pET-28a-IslA-R189E	This Paper	N/A
pET-28a-IslA-R189E/R678E	This Paper	N/A
pET-28a-IslA-I192A	This Paper	N/A
pET-28a-IslA-Q193A	This Paper	N/A
pET-28a-IslA-W374F	This Paper	N/A
pET-28a-IslA-W374Y	This Paper	N/A
pET-28a-IslA-C468S	This Paper	N/A
pET-28a-IslA-E470Q	This Paper	N/A
pET-28a-IslA-R678E	This Paper	N/A
pET-28a-IslA-V680A	This Paper	N/A
pET-28a-IslA-F682A	This Paper	N/A
pET-28a-IslA-F682Y	This Paper	N/A
pET-28a-IslA-G805A	This Paper	N/A

**Software and algorithms**		

GraphPad Prism	GraphPad Software Inc	https://www.graphpad.com/scientific-software/prism/
MATLAB	The MathWorks Inc	https://www.mathworks.com/products/matlab.html
EasySpin	Stoll and Schweiger, 2006	http://www.easyspin.org/
HKL2000	HKL Research, Inc.	https://hkl-xray.com/
Phenix	Adams et al., 2010	https://www.phenix-online.org/
PyMOL	Schrödinger	RRID:SCR_000305

**Other**		

Avogadro	Developed by Geoffrey Hutchison	https://avogadro.cc/
ORCA	Developed by Frank Neese	https://orcaforum.kofo.mpg.de/app.php/portal

### Resource availability

#### Lead contact

Further information and requests for resources and reagents should be directed to and will be fulfilled by the Lead Contact, Catherine Drennan (cdrennan@mit.edu).

#### Materials availability

Plasmids generated in this study will be made available upon request.

#### Data and code availability

The structural data sets generated in this study are available at the Protein Data Bank (PDB IDs: 7KQ3 Ise-bound structure and 7KQ4 glycerol-bound structure). The published article includes all biochemical data generated and analyzed in this study.

### Expermental model and subject details

#### Bacterial culturing conditions

*E. coli* TOP10 and *E. coli* BL21(DE3) cultures were grown at 37°C in Luria-Bertani (LB) broth. Induction of protein expression for *E. coli* BL21(DE3) cultures took place at 15°C. *E. coli* BL21(DE3) ΔiscR cultures were grown at 37°C in LB broth supplemented with glucose (1% w/v), Fe(III)-ammonium-citrate (2 mM), cysteine (2 mM) and sodium fumarate (20 mM). Induction of protein expression took place at 15°C under N_2_ atmosphere.

### Method details

#### Chemicals

All chemicals and reagents were of the highest purity available and purchased from Sigma-Aldrich unless otherwise indicated. Luria-Bertani (LB) medium was obtained from Alfa Aesar. Isopropyl β-D-1-thiogalactopyranoside (IPTG) was purchased from Teknova. NADH and glycerol were purchased from VWR. N-(9-acridinyl)maleimide was purchased from TCI America. SDS-PAGE gels were purchased from Invitrogen. Crystallization reagents were purchased from Hampton Research.

#### Plasmid construction

The wildtype pET-28a-IslA and pET-29b-IslB plasmids were prepared as described previously (Peck et al., 2019). Site-directed mutagenesis of the gene encoding IsIA was performed one of two ways using the corresponding oligonucleotides listed in Table S4. For Q193A and C468S, overlap extension PCR was performed (Heckman and Pease, 2007). For the majority of constructs, PCR reactions of 25 μL contained 12.5 μL of Phusion High-Fidelity PCR Master Mix (New England Biolabs), 50 ng of pET-28a-IslA template, 0.5 μL DMSO and 0.25 μM of each primer. Thermocycling was carried out in a C1000 Gradient Cycler (Bio-Rad) using the following parameters: denaturation for 2 min at 98°C, followed by 22 cycles of denaturation for 30 s at 98°C, annealing for 30 s at 55–65°C (depending on construct), and extension for 8 min at 72°C, followed by a final extension for 10 min at 72°C. Digestion of the methylated template plasmid was performed with Dpn1 (NEB), and 2 μL of each digestion was used to transform 50 μL chemically competent *E. coli* TOP10 cells by incubating them on ice for 2 min, incubating the cells and DNA at 42°C for 30 s, and recovering on ice for 1 min; LB medium (500 μL) was added and the cells were incubated at 37°C for 1.5 hr. The cells were plated on LB supplemented with kanamycin (50 μg/mL, hereafter referred to as LB-Kan50) and then grown at 37°C overnight. Individual colonies were inoculated into 5 mL LB-Kan50 and grown overnight at 37°C. The plasmids were isolated using an E.Z.N.A. Plasmid Mini Kit I (Omega Bio-tek). The identities of each of the resulting plasmids were confirmed by sequencing the purified plasmid DNA (Eton Biosciences).

#### Protein expression and purification

The expression host *E. coli* BL21(DE3) ΔiscR for expression of IslB was constructed as described previously (Peck et al., 2019). Proteins were purified as described previously, with modifications noted below (Peck et al., 2019). For heterologous overexpression, 50 ng of plasmid was transformed into 50 μL chemically competent *E. coli* BL21(DE3) (for IslA), or chemically competent *E. coli* BL21(DE3) ΔiscR (IslB) as above. Cells were plated on LB-Kan50 and grown overnight and single colonies were inoculated into 25 mL LB-Kan50.

For expression of IslA and IslB, a 25 mL starter culture was inoculated into 2 L LB-Kan50 in a 4 L shake-flask for IslA, or into 2 L LB-Kan50 in a 2.8 L baffled screw top flask for IslB. IslB medium was supplemented with glucose (1% w/v) and Fe(III)-ammonium-citrate (2 mM). The cultures were grown at 37°C until they reached an OD_600_ of ∼0.6 and IPTG (0.3 mM) was added. The temperature was lowered to 15°C and the cultures incubated overnight. At the point of induction, the cultures expressing IslB were additionally sparged with N_2_ for 20 min, and cysteine (2 mM) and sodium fumarate (20 mM) were added, before the cultures were sealed with screw-cap tops and electrical tape and incubated overnight at 15°C without shaking.

For the preparation of IslB, all subsequent steps took place at 4°C in an anoxic chamber unless otherwise specified (centrifugation and incubation on a nutator). After overnight growth, the cells were harvested by centrifugation (6,770 x g, 10 min). The supernatant was decanted, and the cells were resuspended in 35 mL lysis buffer. For IslA, the lysis buffer was 50 mM HEPES pH 7.5, 200 mM NaCl, 20 mM imidazole, and for IslB the same buffer was supplemented with lysozyme (8 mg), half of an EDTA-free protease inhibitor tablet and DTT (5 mM). For IslA, the cells were lysed by sonication with a ½” horn (6 min total sonication, 10 s on, 30 s off, 25% amplitude, Branson Ultrasonics). The lysates were clarified by centrifugation (30 min, 20,000 x g). For IslB, the cells were first incubated with the lysozyme at 4°C for 1 hr then lysed by sonication with a ½” horn (7 min total sonication, 10 s on, 30 s off, 25% amplitude), and the lysates were clarified by centrifugation (20,000 x g, 30 min).

The supernatant was incubated with 3 mL Ni-NTA resin (Qiagen) that had been equilibrated with 10 column volumes of the respective lysis buffer for 1 hr. The resin was pelleted (500 x g, 5 min), the supernatant was decanted, and the resin was transferred into a column. After the flowthrough was collected, the resin was washed with 50 mL lysis buffer. The proteins were eluted by sequential washes with elution buffer (50 mM HEPES pH 7.5, 200 mM NaCl, 250 mM imidazole); for IslA, this elution was one step with 12 mL, for IslB three steps of 4 mL each. SDS-PAGE was used to identify the fractions containing the proteins and their purity. Purified proteins were loaded into a dialysis cassette of an appropriate size; 20 kDa MWCO for IslA, and 10 kDa for IslB (Thermo Fisher Scientific).

For endpoint assays, EPR, kinetic analysis and labeling studies, the proteins were dialyzed three times against 1.3 L dialysis buffer (50 mM HEPES pH 7.5, 50 mM NaCl, 10% (v/v) glycerol) for two 2 hr steps and one overnight step. For IslA used in crystallography, the dialysis buffer was modified to not include glycerol (50 mM HEPES pH 7.5, 50 mM NaCl). The dialyzed protein solution was concentrated via centrifugation in a 20 mL 30 kDa centrifuge filter (IslA) or 6 mL 10kDa centrifuge filter (IslB) (3,220 x g, 20 min spins) until the desired concentration was reached. Finally, all proteins were aliquoted into cryovials fitted with an O-ring, flash frozen in liquid N_2_, and stored at −80°C. The cryovials with IslB were sealed in anoxic Hungate tubes (ChemGlass) before freezing.

Recombinant enzymes used for enzymatic assays were handled in an anoxic vinyl chamber (Coy Laboratories) (97% N_2_/3% H_2_ atmosphere). Samples were routinely rendered anoxic as follows. Consumable goods were brought into the glovebox the day before being used. Solid chemicals were brought into the anoxic chamber in Eppendorf tubes that had been perforated. Protein solutions were either purified and stored under anoxic conditions (IslB), or rendered anoxic before use by transfer to amber LC-MS vials on ice that were sealed with septa and N_2_ was passed over the headspace for 15 min before being brought into the anoxic chamber (IslA). Buffer components were routinely rendered anoxic by sparging them with N_2_ prior to use.

#### Crystallization of IslA from *B. wadsworthia*

Initial screening was performed with the aid of an Art Robbins Phenix micro-pipetting robot and Formulatrix Rock Imager; initial crystallization conditions of a well solution containing 200 mM calcium acetate and 20% w/v PEG 3350 were found using the Hampton PEG/ION HT screen. Optimized crystals of glycerol-bound IslA from *Bilophila wadsworthia* were grown aerobically by hanging drop vapor diffusion at 22°C. 1 μL of unactivated IslA protein with intact N-terminal His-tag (7.5 mg/mL in a buffer containing 50 mM HEPES pH 7.5, 50 mM NaCl, 10% (v/v) glycerol and 3 mM Isethionate) was mixed with 1 μL of an optimized precipitant solution (200 mM calcium acetate and 15% w/v PEG 3350) in a sealed well with 500 μL of precipitant solution. Crystals grew after 2 weeks and were transferred in three steps of increasing glycerol concentration into a final cryogenic solution containing the precipitant solution supplemented with 20% (v/v) glycerol and flash frozen in liquid nitrogen.

Crystals of isethionate-bound IslA grew after 2 months, aerobically, by hanging drop vapor diffusion at 22°C. 1 μL of unactivated IslA protein with intact N-terminal His-tag (7.5 mg/mL in a buffer containing 50 mM HEPES pH 7.5, 50 mM NaCl, and 30 mM sodium isethionate) was mixed with 1 μL of precipitant solution (0.16 M NaBr and 20% PEG 3350) in a sealed well with 500 μL of precipitant solution. Crystals were cryoprotected with paraffin oil and flash frozen in liquid nitrogen.

#### Structure determination of IslA

A native dataset of IslA was collected at the Advanced Photon Source (Argonne, IL) on beamline 24ID-C using the Pilatus-6M pixel array detector at a temperature of 100 K and wavelength of 0.9792 Å (12,662 eV). Data were indexed, integrated and scaled in HKL2000 (Otwinowski and Minor, 1997) in the space group P2_1_2_1_2 to 2.26 Å resolution (see Table 1, below).

The structure of IslA was solved by molecular replacement in Phaser (Mccoy et al., 2007) using chain A of the structure of CutC from *Desulfovibrio alaskensis* (PDB 5FAU, 34.4% identity) (Bodea et al., 2016) after trimming side chains non-identical to IslA with Sculptor (Bunkoczi and Read, 2011). A solution with two IslA monomers, each forming a physiological dimer by crystallographic symmetry, were found (LLG and TFZ scores of 229.343 and 16.2, respectively), in the asymmetric unit (ASU). An initial round of automated model building and structure refinement was performed using Phenix AutoBuild (Terwilliger et al., 2008) (yielding R_work_ and R_free_ of 29.52% and 33.94%, respectively). After a rigid body refinement of the automated model, the model was extensively rebuilt using iterative steps of manual model building in Coot (Emsley and Cowtan, 2004) and refinement in Phenix (Adams et al., 2010) using atomic coordinates, atomic displacement parameters (B-factors) and two-fold non-crystallographic symmetry (NCS) restraints, without sigma cutoffs. Water molecules were added and verified manually in later stages of refinement using F_o_-F_c_ electron density map contoured to 3.0σ as criteria. NCS restraints were released in final stages of refinement. Refinement statistics can be found in Table 1.

The final structure of IslA contains 2 chains each with 6–830 (of 830 residues) and a glycerol molecule in the active site. Composite omit maps calculated in Phenix (Adams et al., 2010) were used to validate the model. Model geometry was analyzed using MolProbity (Chen et al., 2010). Ramachandran statistics analyzed by MolProbity (Chen et al., 2010) indicated 97.3%, 2.6%, and 0.1% of residues in the favored, allowed, and disallowed regions, respectively, and 98.7% of residues have favorable rotamers. Ile469 of chain A and B were the only two Ramachandran outliers but best fit the composite omit density. PyMol was used to generate figures (Schrodinger, 2010). Crystallography software packages were compiled by SBGrid (Morin et al., 2013).

#### Structure determination of Ise-bound IslA

A substrate-bound dataset of IslA was collected at the Advanced Photon Source (Argonne, IL) on beamline 24ID-E using the Dectris Eiger 16M pixel array detector at a temperature of 100 K and wavelength of 0.9792 Å (12,662 eV). Data were indexed, integrated and scaled in HKL2000 (Otwinowski and Minor, 1997) in the space group P2_1_2_1_2_1_ to 2.70 Å resolution (see Table 1, below).

The structure of IslA was solved by molecular replacement in Phaser (Mccoy et al., 2007) using chain A of the structure of IslA with glycerol bound after removal of ligands and water molecules. A solution with four IslA monomers, forming a dimer of dimers, were found (LLG and TFZ scores of 14,148.983 and 121.0, respectively), in the asymmetric unit (ASU). After a rigid body refinement of the automated model, the model was extensively rebuilt using iterative steps of manual model building in Coot (Emsley and Cowtan, 2004) and refinement in Phenix (Adams et al., 2010) using atomic coordinates, atomic displacement parameters (B-factors) and two-fold non-crystallographic symmetry (NCS) restraints, without sigma cutoffs. The isethionate ligand parameter files were generated using the eLBOW tool of Phenix (Moriarty et al., 2009), and correct ligand placement was verified using composite omit maps. Water molecules were added and verified manually in later stages of refinement using F_o_-F_c_ electron density map contoured to 3.0σ as criteria. Refinement statistics can be found in Table 1.

The final structure of isethionate-bound IslA contains 4 chains each with 6–830 (of 830 residues) and an isethionate molecule in each active site. Composite omit maps calculated in Phenix (Adams et al., 2010) were used to validate the model. Model geometry was analyzed using MolProbity (Chen et al., 2010). Ramachandran statistics analyzed by MolProbity (Chen et al., 2010) indicated 96.9%, 2.9%, and 0.2% of residues in the favored, allowed, and disallowed regions, respectively, and 99.1% of residues have favorable rotamers. Ile469 of chain A, B, C and D as well as Thr313 of chain B were the only Ramachandran outliers, but best fit the composite omit density. PyMol was used to generate figures (Schrodinger, 2010). Crystallography software packages were compiled by SBGrid (Morin et al., 2013).

#### Generation of glycyl radical in IslA

The GRE was activated as described previously (Peck et al., 2019) in an anoxic chamber by incubating IslB (80 μM), IslA (40 μM), acriflavine (100 μM), *S*-adenosylmethionine (1 mM), and bicine (50 mM pH 7.5) in reaction buffer (50 mM HEPES pH 7.5, 50 mM NaCl) at 25°C for 2 hr in a 275 μL scale for EPR spectroscopy. No substrate (isethionate) was added during activation. The entire activation mixture was then loaded into EPR tubes with 4 mm outer diameter and 8″ length (Wilmad LabGlass), sealed, removed from the anoxic chamber, and slowly frozen in liquid N_2_. Perpendicular mode X-band EPR spectra were recorded on either a Bruker ElexSysE500 EPR instrument equipped with a quartz finger dewar (Wilmad Lab-Glass) for acquiring spectra at 77 K with liquid N_2_ or a Bruker EMX-Plus EPR instrument equipped with a Bruker/ColdEdge 4K waveguide cryogen-free cryostat set at 77K. The samples were acquired with the following parameters on the ElexSysE500 EPR: microwave frequency: 9.41 GHz; power: 20 μW (40 dB attenuation); center field: 3350 Gauss; sweep width: 200 Gauss; conversion time: 20.48 ms; modulation gain: 60 dB modulation gain for samples; 30 dB for external standards; time constant: 20.48 ms; modulation amplitude: 4 G; modulation frequency: 100 kHz. The samples were acquired with the following parameters on the EMX-Plus EPR: microwave frequency: 9.37 GHz; power: 1.262 μW (52 dB attenuation); center field: 3350 Gauss; sweep width: 200 Gauss; conversion time: 41.97 ms; modulation gain: 30 dB; time constant: 0.01 ms; modulation amplitude: 4 G; modulation frequency: 100 kHz. Normalization due to differences in modulation gain were automatically performed by the spectrometer. Typically, only a single scan was recorded on the ElexSysE500 to minimize any disruption due to bubbling from the liquid N_2_, whereas typically 5 scans were recorded on the EMX-Plus. The field was calibrated by using an external standard of bisdiphenylene-β-phenylallyl (BDPA) with g = 2.0026 (Bruker). An external standard of Frémy salt was prepared by dissolving K_2_(SO_3_)_2_NO in either anoxic 0.5 M KHCO_3_ or anoxic 20 mM HEPES pH 7.2. The concentration of the standard was measured by its absorbance at 248 nm (ε = 1,690 M^−1^ cm^−1^) using a NanoDrop 2000 UV-Vis Spectrophotometer. The double integral of the Frémy salt standard was calculated on the EPR spectrometer and then used to determine the concentrations of each of the protein samples from that set of EPR measurements. Frémy salt standards were prepared fresh and run for each set of EPR measurements on either instrument. The EPR spectra from the activation mixtures were simulated using EasySpin in MATLAB using the Levenberg/Marquardt algorithm (Stoll and Schweiger, 2006). Activations were repeated in triplicate to obtain the error bars shown in Table 2.

#### Detection of sulfite for endpoint assays

IslA was first activated as described above for EPR spectroscopy on a 50 μL or 100 μL scale. Activated IslA (5.9 μM total GRE) was then added to reaction buffer (50 mM HEPES pH 7.5, 50 mM NaCl) supplemented with yeast alcohol dehydrogenase (8 μM) and NADH (3 mM) in a 50 μL scale, and the reaction initiated by addition of 10 mM isethionate. The reaction mixture was incubated for 1 hr and was then transferred out of the anoxic chamber and derivatized according to a previously reported procedure (Peck et al., 2019). To distinguish catalytically dead mutants from mutants with minimal activity toward isethionate, a 2 hr incubation with 10 mM isethionate and 11.8 μM total IslA was also performed. The reactions were repeated in quadruplicate. For derivatization, a 100 mL solution of 0.3 M boric acid, 0.3 M KCl, and 0.02 M Na_2_-EDTA was mixed with a 50 mL solution of 0.3 M Na_2_CO_3_ and 0.02 M Na_2_-EDTA to adjust the solution of the mixture to pH 8.8. 150 μL of this solution was added to each reaction, followed by 50 μL of an acetone solution containing N-(9-acridinyl)maleimide (0.1% w/v). Freshly prepared sodium sulfite standards were derivatized at the same time. The reactions were incubated at 37°C for 2 hr in the dark. The fluorescence intensity was recorded using a Synergy HTX Plate Reader (BioTek) with the excitation wavelength of 360 nm and the emission wavelength of 440 nm.

#### Kinetics analysis of isethionate cleavage

IslA was first activated as described above for EPR spectroscopy on a 250 μL scale. Activated IslA (0.8–4.5 μM total IslA depending on mutant activity) was mixed with yeast alcohol dehydrogenase (2 μM) and NADH (200 μM) on a 200 μL scale in a 96-well plate, as described previously (Peck et al., 2019). The reactions were initiated by addition of isethionate (1–50 mM), and the plate was loaded into a PowerWave HT plate reader (BioTek) set to 30°C. The pathlength-corrected absorbance at 340 nm was recorded every 10 s for up to 30 min. The observed rate constant was fit to the standard Michaelis-Menten steady-state equation (*k*_*obs*_ = *k*_*cat*_∗ [S]/(*K*_*M*_ + [S])) in Graphpad Prism 8.0.1. Reactions were done in triplicate to obtain the error bars shown in Table 2.

#### Synthesis of 2,2-d_2_-isethionate

**Figure:**
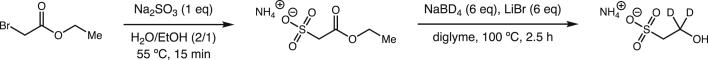

The deuterated substrate was prepared using a previously reported procedure (Harmer et al., 2005).

**Figure:**
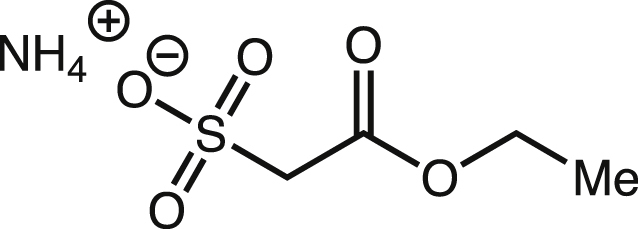

Ammonium ethyl sulfoacetate. Under air, a 50-mL round-bottom flask (rbf) was equipped with a magnetic stir bar, then charged with sodium sulfite (2.5 g, 20 mmol, 1 equiv.) and distilled water (8 mL). The mixture was sonicated to yield a clear solution then it was cooled to 0°C with stirring. A solution of ethyl bromoacetate (2.2 mL, 3.3 g, 20 mmol) in absolute ethanol (4 mL) was added over 5 min. Precipitate formed heavily by the end of the addition process. The mixture was heated to 55°C for 15 min. Most precipitate dissolved during the heating period. While hot, the mixture was decanted to remove residual precipitate. The clear solution was cooled then concentrated to yield a waxy solid.

The resulting solid was suspended in a hot solution of AcOH/EtOAc (2/1 ratio, 18 mL, ∼ 60°C). The mixture was swirled at 60°C for about 10 min, then it was quickly filtered over Celite. Once the solution was cooled to room temperature, EtOAc (50 mL) was added, which resulted in a white precipitate. The precipitate was separated by centrifugation. The solid was resuspended in EtOAc and separated again by centrifugation. This washing procedure was repeated two more times. The precipitate was dissolved in distilled water (3 mL). The pH of the solution was approximately 3. Amberlite IR120 resin (Oakwood) was slowly added to adjust to the pH to 1. The mixture was decanted to remove the resin. The resulting solution was cooled on an ice bath, then cooled concentrated ammonium hydroxide (Avantor) was added to adjust the pH to 7. The final solution was concentrated by lyophilization to yield an off-white solid.

The off-white solid was dissolved in ethanol (2 mL), then it was filtered with a syringe filter. Diethyl ether (20 mL) was slowly added, which resulted in a white precipitate. The solid was isolated via filtration and dried under hi-vac overnight. NMR data is in agreement with previously reported data. According to the literature, ∼35% by weight of the crude solid is ammonium ethyl sulfoacetate. Since the next step utilizes excess reagents (>10 equiv.), we assumed a 35% by weight for stoichiometry calculation. The procedure yielded 1.5 g product (35% w/w, 14% yield). ^1^H NMR (D_2_O, 400 MHz): δ 1.31 (t, *J* = 7.1z Hz, 3H), 3.98 (s, 2H), 4.27 (q, *J* = 7.2 Hz, 2H).

**Figure:**
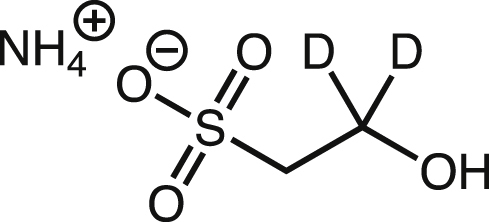

Ammonium [2,2-^2^H_2_]-2-Hydroxyethanesulfonate. Under nitrogen, a two-neck 100-mL rbf, equipped with a reflux condenser and a magnetic stir bar, was charged with sodium borodeuteride (1.1 g, 26 mmol, 13 equiv., Acros Organics) and diglyme (10 mL). The mixture was stirred for 15 min at room temperature before lithium bromide (2.3g, 26 mmol, 13 equiv.) was added. This mixture was stirred for another 30 min at room temperature. Ammonium ethyl sulfoacetate (35% by weight, 1.1 g, 2.0 mmol) was added then the mixture was heated at 100°C for 2.5 hr under nitrogen.

The mixture was cooled to room temperature then quenched by slow addition of MeOH (∼20 mL total) and distilled water (∼20 mL). MeOH and water were added dropwise to limit gas evolution during the quenching process. The pH of the final solution was about 11. Amberlite IR120 resin (Oakwood) was added to adjust the pH to 1. This resulted in a yellow solution. The resin was removed by decanting, then 50 mL of MeOH was added before the solution was concentrated to yield an oily mixture. This mixture was resuspended in 50 mL of MeOH and the mixture was concentrated. This process was repeated 5 more times, which resulted in a sticky solid. The solid was dissolved in water (2 mL) and cooled on an ice bath. Cooled concentrated ammonium hydroxide was added to adjust the pH to 7. The final solution was concentrated by lyophilization to yield a light brown solid.

The light brown solid was dissolved in 5 mL of methanol, then the mixture was filtered with a syringe filter. Diethyl ether (about 30 mL) was added, which resulted in a white precipitate. The precipitate was isolated by filtration, then dried on hi-vacuum overnight.

The product was further purified by dissolving in water (1 mL), and then ethanol (4 mL) was added. The mixture was allowed to sit at 4°C overnight. The precipitate was filtered then the solution was concentrated to yield the final product as a white solid (110 mg, 37% yield). NMR data is in agreement with previously reported data. ^1^H NMR (D_2_O, 400 MHz): δ 3.18 (s, 2H).

#### Detection of deuterium-labeled ethanol

IslA was first activated on a 250 μL scale as described above for EPR spectroscopy. After the 2 hr incubation, a boiled enzyme control was prepared by incubating the activation mixture at 95°C for 10 min in a C1000 Gradient Cycler (Bio-Rad). Either active IslA or boiled IslA (1.6 μM total IslA) was mixed with NADH (3 mM), yeast alcohol dehydrogenase (8 μM) and either unlabeled or 2,2-d_2_-isethionate (2 mM) in reaction buffer (50 mM HEPES pH 7.5, 50 mM NaCl) on an 800 μL scale. The reactions were set up in triplicate. Yeast alcohol dehydrogenase was used in these assays to prevent accumulation of acetaldehyde that has been shown to recombine with sulfite (Peck et al., 2019). The reaction mixtures were left in the anoxic chamber overnight. After overnight incubation, they were removed from the chamber and immediately added to a 10 mL headspace vial with 4.2 mL of water and 1.7 g NaCl and sealed tightly. Vials were stored at 4°C prior to GC-MS analysis.

Headspace gas chromatography-mass spectrometry (GC-MS) experiments were conducted on the TRACE 1310 Gas Chromatograph with a Q Exactive GC Orbitrap. Headspace extractions were performed at 85°C with agitation for 10 min on an autosampler (Thermo Scientific TriPlus RSH). A transfer syringe held at 120°C was used to inject 1 mL of headspace sample into the instrument. The column used was a fused-silica capillary column of cross-linked DB-624UI (30 m × 0.32 mm × 1.80 μm, Agilent). The inlet helium carrier gas flow rate was 2.3 mL/min. For spectra collected with positive chemical ionization (PCI), the conditions were as follows: split ratio of 20; oven temperature program 30°C for 3 min, 50°C/min to 250°C, hold for 3 min; MS transfer line at 220°C; CI gas type methane with 2 mL/min flow rate; ion source temperature 120°C; full MS-SIM from 1–7 min in positive polarity; resolution 120,000; AGC target 1e6; scan range 30–100 m/z; max IT auto. For spectra collected with electron impact (EI) ionization the conditions were: split ratio of 20; oven temperature program 30°C for 3 min, 50°C/min to 250°C, hold for 3 min; MS transfer line at 220°C; ion source temperature 200°C; full MS-SIM from 1–7 min in positive polarity; resolution 120,000; AGC target 1e6; scan range 30–100 m/z; max IT auto. The retention time of the ethanol peak was 2.03–2.07 min. The entire peak was extracted with background correction to generate the displayed mass spectra. The relative intensity is scaled to the maximum intensity in the plotted range of m/z values. Data was analyzed using Thermo Xcalibur Qual Browser 3.0.63.

#### Small molecule QM calculations

Electronic structure calculations were performed to investigate the energetics of representative models of the isethionate H abstraction by Cys radical. The cysteine radical was modeled as CH_3_S⋅ and glutamate, where present, was modeled as acetate. Fully optimized structures of the reactant substrates and transition states (TSes) were obtained using ORCA (Neese, 2012) v.4.0.1.2 in implicit solvent models but in the absence of the explicit enzyme environment. Free gas-phase geometry optimizations of substrates and TSes were performed using hybrid (B3LYP (Anandakrishnan et al., 2012; Bayly et al., 1993; Becke, 1993)) density functional theory (DFT) with the 6-31G∗ basis set (Ditchfield et al., 1971). Frequency calculations were performed on the optimized geometries at the same level of theory, i.e., B3LYP/6-31G∗, and thermodynamic corrections were obtained.

The geometry optimizations of substrates and TSes were carried out in redundant internal coordinates using the BFGS and Bofill algorithms, respectively, with default thresholds of 3x10^−4^ hartree/bohr for the maximum gradient and 5x10^−6^ hartree for SCF convergence. Initial structures of intermediates were built by hand in Avogadro (Hanwell et al., 2012) v1.20, and TSes were modified from the optimized intermediates by stretching the forming and breaking bonds. All initial and optimized geometries are provided in the Supplemental Information as Data S1.zip and Data S2.zip file, respectively. Both.zip files are related to Figure 6.

Numerical Hessian calculations were carried out where the Hessian was computed using the central differences approach after 6*N* displacements (where *N* is the number of atoms in a given system). The presence of a single imaginary frequency was confirmed for both the TSes corresponding to the hydrogen atom transfer from isethionate to CH_3_S⋅ in the presence and absence of acetate, while the substrates had no imaginary frequencies indicating that the converged geometries of substrates corresponded to energy minima.

Thermochemistry properties such as inner energy (*U*), enthalpy (*H*), entropy (*S*) and the Gibbs free energy (*G*) were then computed at 298.15 K and 1 atm for these gas-phase models using statistical mechanics (Table S1). Single point energy calculations were carried out on the optimized geometries at the domain-localized pair natural orbital coupled cluster single doubles and perturbative triples (DLPNO-CCSD(T) (Riplinger and Neese, 2013)) level of theory using tight PNO thresholds (Table S2). Dunning-style correlation consistent double-ζ and triple-ζ (i.e., aug-cc-pVDZ and aug-cc-pVTZ) basis sets were employed to enable two-point extrapolation (Helgaker et al., 1997; Neese and Valeev, 2011; Zhong et al., 2008) to the complete basis set (CBS) limit. Since implicit solvent models are not implemented in DLPNO-CCSD(T), the gas phase DLPNO-CCSD(T) energies were corrected with the conductor-like polarizable continuum model (Barone and Cossi, 1998) (C-PCM) solvation energies in combination with the conductor-like screening solvent model (COSMO) epsilon function type obtained at the MP2/CBS level of theory in ORCA (Table S2). The solvent corrections were computed as the difference between gas-phase MP2/CBS single point energies and solvent-corrected MP2/CBS single point energies. The solvent corrections were carried out with two dielectric values, ε = 10 and 78.39, approximately mimicking the protein and an aqueous environment, respectively. The solvent-corrected DLPNO-CCSD(T)/CBS energies were used in combination with the thermodynamic corrections to predict the value of *G*^*0*^ at a temperature of 298.15 K and pressure of 1 atm.

### Quantification and statistical analysis

Statistical analysis was performed using either GraphPad Prism or Microsoft Excel. Statistical details of the experiments can be found in the corresponding figure or table legends, and are mentioned in the STAR Methods.

## Supplemental information

Document S1. Figures S1–S9 and Tables S1–S4

Data S1. Initial geometries for QM calculations

Data S2. Optimized geometries from QM calculations

Document S2. Article plus supplemental information
