# Supplementary material for: Molecular basis of C-S bond cleavage in the glycyl radical enzyme isethionate sulfite-lyase
Source: Cell Chem Biol. 2021 Sep 16;28(9):1333–1346.e7. doi: 10.1016/j.chembiol.2021.03.001 (PMC8473560; doi:10.1016/j.chembiol.2021.03.001)
Supplement: Document S1. Figures S1–S9 and Tables S1–S4 [file mmc1.pdf]

Cell Chemical Biology, Volume *28*

Supplemental information

Molecular basis of C-S bond cleavage in the glycyl radical enzyme isethionate sulfite-lyase

Christopher D. Dawson, Stephania M. Irwin, Lindsey R.F. Backman, Chip Le, Jennifer X. Wang, Vyshnavi Vennelakanti, Zhongyue Yang, Heather J. Kulik, Catherine L. Drennan, and Emily P. Balskus

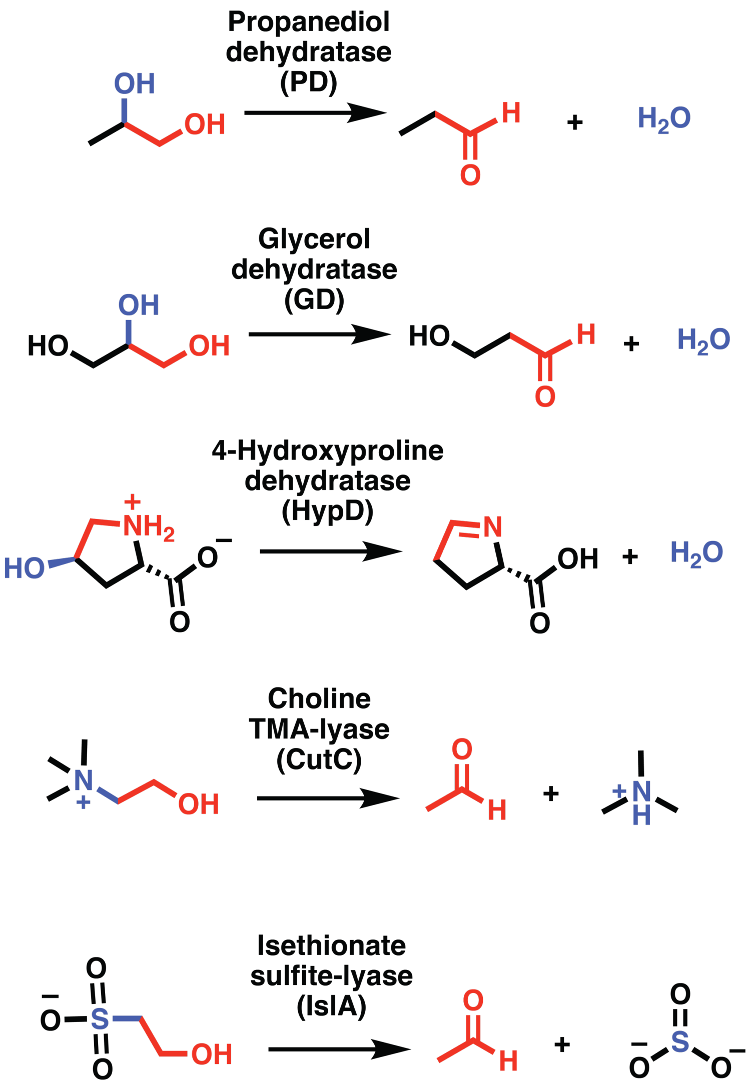

Fig. S1, related to Figure 1: GRE reaction schemes. Eliminated functional groups and bonds that undergo cleavage are shown in blue. Moieties undergoing oxidation to drive elimination are shown in red.

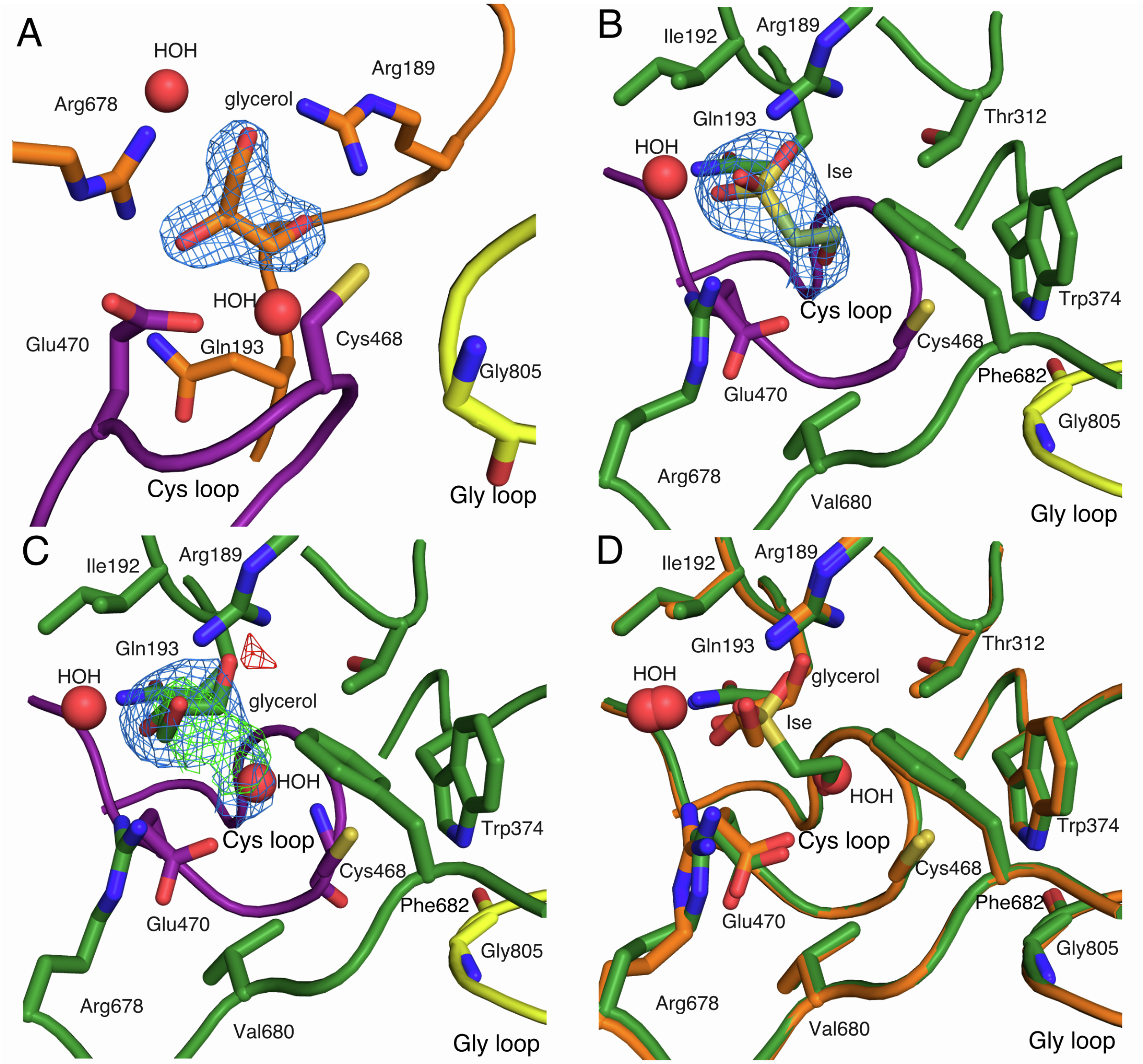

Fig. S2, related to Figure 2: Comparison of the glycerol-bound and Ise-bound IslA structures. (A) $2F_o$-F_c composite omit map contoured to 1.5 σ around glycerol. IslA is shown in orange with Gly loop and Cys loop shown in yellow and purple, respectively. Water molecules are shown as red spheres. (B) Isethionate-bound structure with $2F_o$-F_c composite omit map contoured to 1.5σ (blue) and with F_o-F_c map contoured to +3σ (bright green) or to -3σ (red). (C) Glycerol and a water molecule refined into active site density of isethionate-bound structure with $2F_o$-F_c composite omit map contoured to 1.5σ and with F_o-F_c map contoured to +3σ (bright green) or to -3σ (red). (D) Overlay of glycerol-bound IslA (orange) and isethionate-bound IslA (green).

		β1	β2	β3	Cys Loop	β8	Gly Loop
		189 193	312	374	468	678 682	805
	TdcE: WP_000861734.1	PDGYGRGRIIG	N-GGAMS	DPI-W------ATE	IACCVSPMV	PTQSILTITSN	RVSGYAV
	PFl: WP_001292822.1	PDAYGRGRIIG	N-GAAMS	DPI-W------ATE	IACCVSPMI	PTQSVLTITSN	RVSGYAV
	HPAD: WP_029163539.1	----PQGREVI	DPQSGLS	NTF--------NNL	LGGCLESAP	LYLCQISVSTH	RVAGFTQ
	BSS: AAK50372.1	----GYNSIVP	RYA-SGY	YREIFPGSNDLFIL	NVLCMSPGL	VLPTGQAVGLY	RVSGYSA
	PAD: AVQ67923.1	---DMGGMVVP	CMTVHHA	LAI-WQQSRIAQQL	VAGCVQSII	ITPSAYSVTAH	RVAGFSA
	AEG16045.1	ASIRNSLQWVH	CIGAVVG	YSH-W------EAL	ASGCSEVRM	LDVRYVPVTAH	RVAGYSA
	WP_087350898.1	ASWRSSLQWVH	RTGTTIS	YAH-W------EAV	ASGCTETRM	VDTRYVPVTAH	RVAGYSA
	ORT99124.1	SSMRSALQWVH	KTGATIS	YAH-W------EAV	VSGCTEVRM	IDLRYVSQSAN	RVAGYSA
	WP_015774320.1	SSYRSALQWVP	KASAIIS	YAH-W------EAV	VSGCTEARM	VDVRYVPITSH	RIAGYSA
	WP_066178498.1	ASFRSSLQWVH	KTGTIIS	YAH-W------EAV	VSGCAECRM	LDLRYVPFTSH	RIAGYSA
	WP_043166045.1	ASFRSSLQWVH	KTGTIIS	YAH-W------EAV	VSGCAECRM	MDIRLVPFTSH	RIAGYSA
	EKY23466.1	ASFRSSLQWVH	KTGTIIS	YAH-W------EAV	VSGCAECRM	MDLRLVPFTSH	RIAGYSA
	WP_087193818.1	ASFRSSNQWVH	KTGTIIS	YAH-W------EAV	VSGCAEARM	YCLRYVPFTSH	RIAGYSA
	WP_094606507.1	ATMRSALQWVH	KTGATIS	YAH-W------EAV	VSGCTEVRM	LDVRYVPVTSH	RVAGYSA
IslAs	WP_073616382.1	SSFRSSIQWVH	KTGTIIS	YAH-W------EAV	VSGCSESRM	LDLRLVPFTSH	RIAGYSA
	WP_042216496.1	ASFRSSIQWVH	KTGTVIS	YAH-W------EAV	VSGCAECRM	LDLRYVPFTSH	RIAGYSA
	WP_071982924.1	ASFRSSIQWVH	KTGTIIS	YAH-W------EAV	VSGCAECRM	LDLRYVPFTSH	RVAGYSA
	SKA70293.1	SSFRSSLQWVH	KTGTIIS	YAH-W------EAV	VSGCAEIRM	LDLRLVPFTSH	RIAGYSA
	WP_009733371.1	SSFRSSIQWVH	KTGTTIS	YAH-W------EAV	VSGCIEIRM	NDARYVPFTSH	RIAGYSA
	WP_074216573.1	SSFRSSIQWVH	KTGTIVS	YAH-W------EAV	VSGCTEARM	NDVRYVPFTSH	RIAGYSA
	WP_041724859.1	SSFRSSIQWVH	KTGTTIS	YAH-W------EAV	VSGCTEARM	NDVRYVPFTSH	RIAGYSA
	WP_028577862.1	ASFRSSIQWVH	KTGTTIS	YAH-W------EAV	VSGCTEARM	NDVRYVPFTSH	RIAGYSA
	WP_015731066.1	SSFRSSIQWVH	KTGTIIS	YAH-W------EAV	VSGCAECRM	LDLRLVPFTSH	RIAGYSA
	SCM78658.1	ASFRSSIQWVH	KTGTIIS	YAH-W------EAV	VSGCSECRM	LDLRLVPFTSH	RIAGYSA
	WP_015942724.1	ASFRSSIQWVH	KTGTIIS	YAH-W------EAV	VSGCAECRM	LDLRLVPFTSH	RVAGYSA
	WP_073041932.1	SSFRSSLQWVH	KTGTIIS	YAH-W------EAV	VSGCAEVRM	LDLRYVPFTSH	RIAGYSA
	CutC: WP_011369019.1	-----GGDSNP	N-QTGMS	YQP-F------VNM	LMGCVEPQK	LSHGTLSISNN	RVAGYSA
	HypD: WP_021363977.1	-----APGHTV	NIWDAFT	YTD-F------ANI	TSGCVETGC	YRVDMLPTTCH	RVAGYSD
	PD: WP_007885173.1	-----VGHVTV	S-GHSIS	YSL-F------QNL	IIGCVEPQV	YQAGLYPVSAN	RVAGYSA
	GD: WP_002581613.1	-----VGHVSV	N-GHSIS	YPM-Y------QNL	IIGCVEPQK	FQPGLYPSSIN	RVAGYSA

Fig. S3, related to Figure 3: A Multiple sequence alignment for key structural regions for putative IslAs and several characterized GREs. NCBI Accession codes are listed for each sequence, numbered as residues in IslA from *B. wadsworthia* (WP_009733371.1). Residues conserved in all GREs are show in yellow. Residues conserved in GRE eliminases are shown in orange. Residues conserved in IslAs are shown in green. Sequences were aligned using Clustal Omega (Sievers et al., 2011).

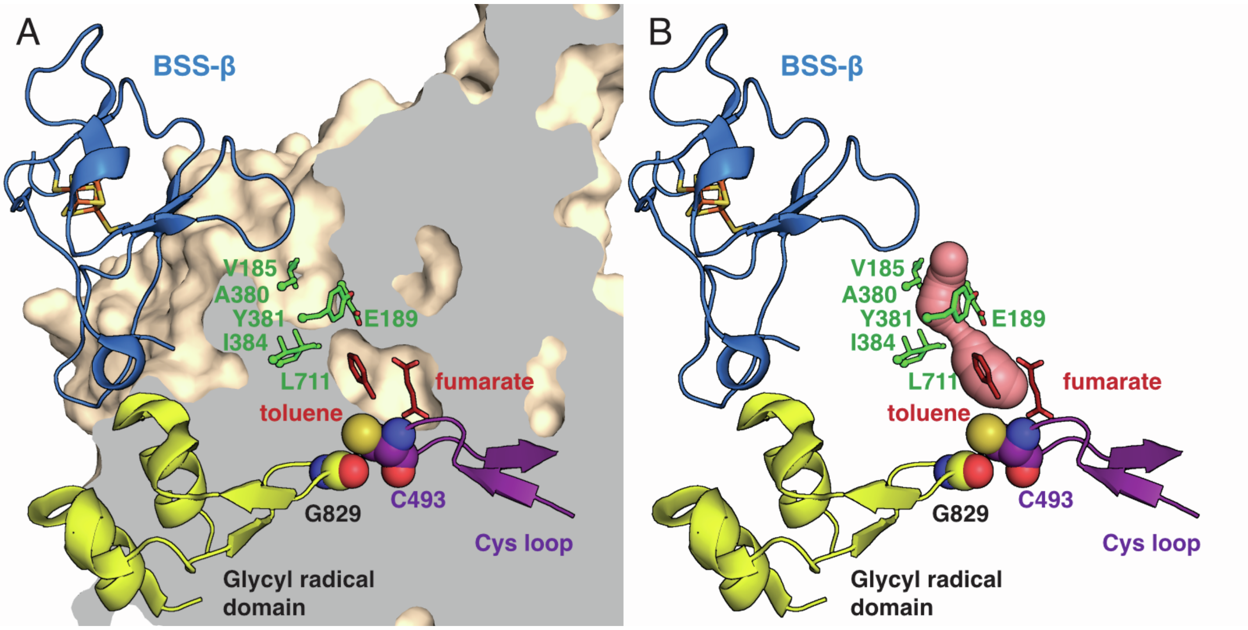

Fig. S4, related to Figure 5: Channel views and conservation in GREs. (A) Van der Waals surface of BSS (PDB ID: 5BWE) is shown as a cutaway with substrates fumarate and toluene (red) above the glycyl radical domain (yellow) and Cys loop (purple). The channel contains a bottle neck created by the residues shown in green and is capped by BSS-β (blue) (B) The substrate channel (pink) of BSS between the surface of the protein and fumarate-binding site as generated by CAVER 3.0 (Chovancova et al., 2012) using the BSS-αβγ structure with BSS-β removed.

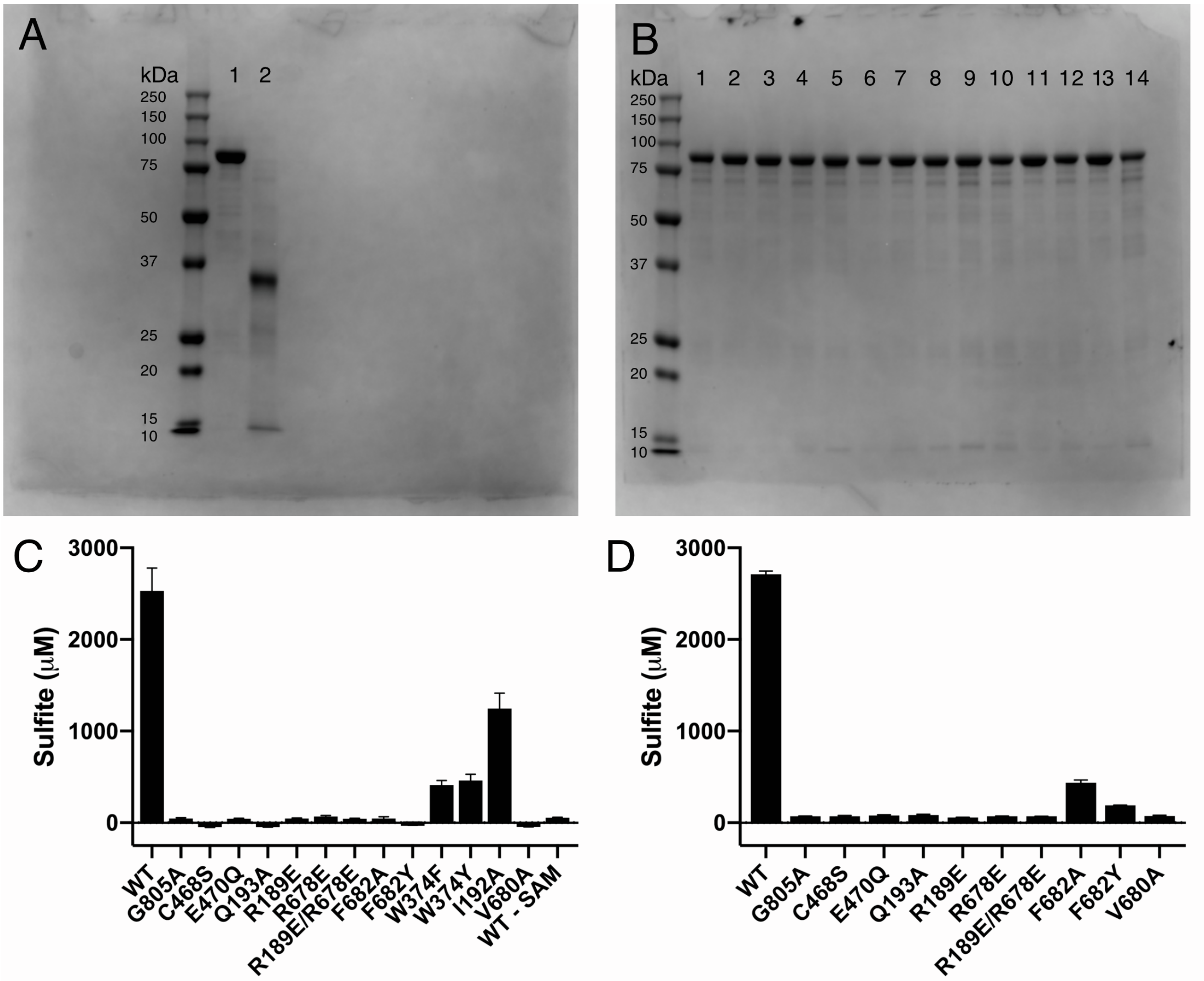

Fig. S5, related to Table 2: SDS-PAGE of recombinant enzymes purified for this study and sulfite endpoint detection demonstrates IslA variant activity toward isethionate. (A) Precision Plus Protein All Blue Standards (BioRad), IslA-WT (lane 1), and IslB (lane 2). (B) Precision Plus Protein All Blue Standards (BioRad), IslA-WT (lane 1), IslA-R189E (lane 2), IslA-R189E/R678E (lane 3), IslA-I192A (lane 4), IslA-Q193A (lane 5), IslA-W374F (lane 6), IslA-W374Y (lane 7), IslA-C468S (lane 8), IslA-E470Q (lane 9), IslA-R678E (lane 10), IslA-V680A (lane 11), IslA-F682A (lane 12), IslA-F682Y (lane 13), IslA-G805A (lane 14). (C) Initial assay conditions with a 1-hour incubation of activated GRE with isethionate demonstrated sulfite production for WT, W374F, W374Y and I192A. (D) A 2-hour incubation with twice the enzyme concentration shows that F682A and F682Y are also capable of generating sulfite from isethionate. All assays were repeated in quadruplicate. Bars represent the mean ± standard deviation of the replicates.

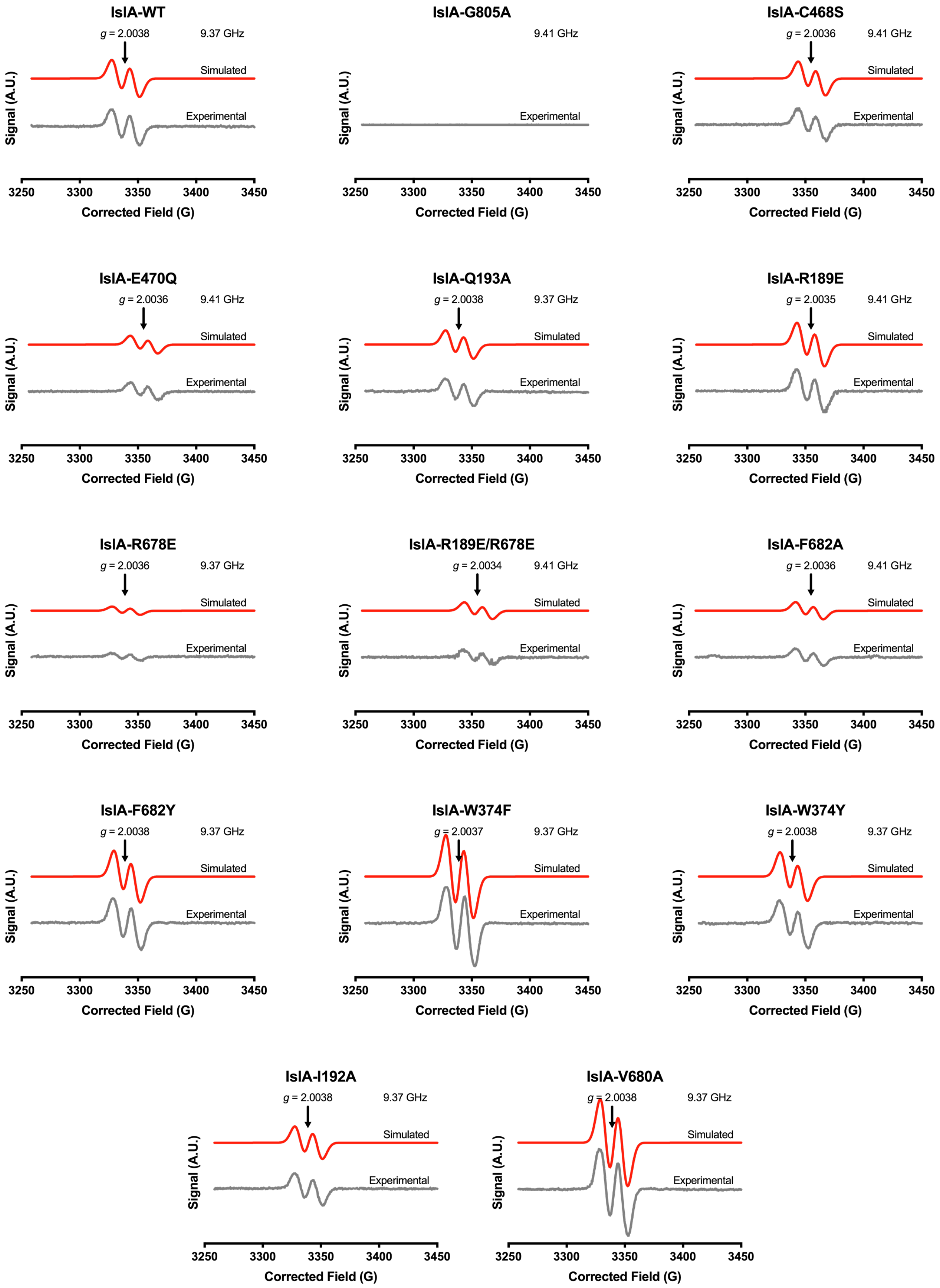

Figure S6, related to Table 2: Representative EPR spectra of IslA variants. To account for differences in signal (A.U.) between instruments, the signal for each experiment has been scaled, after a baseline-correction to set the background signal to 0, such that the maximum

amplitude is set to the value of radical installation, as determined by using a Frémy standard run on the same day as the respective sample. The G805A trace was not scaled due to no detected radical signal. The IslA-WT, IslA-Q193A, IslA-R678E, IslA-F682Y, IslA-W374F, IslA-W374Y, IslA-I192A and IslA-V680A spectra were collected on the EMX-Plus EPR with microwave frequency: 9.37 GHz; power: 1.262 µW (52 dB attenuation); conversion time: 41.97 ms; modulation gain: 30 dB; time constant: 0.01 ms; modulation amplitude: 4 G; modulation frequency: 100 kHz. The IslA-G805A, IslA-C468S, IslA-E470Q, IslA-R189E, IslA-R189E/R678E, and IslA-F682A spectra were collected on the ElexSysE500 EPR with microwave frequency: 9.41 GHz; power: 20 µW (40 dB attenuation); conversion time: 20.48 ms; modulation gain: 60 dB; time constant: 20.48 ms; modulation amplitude: 4 G; modulation frequency: 100 kHz.

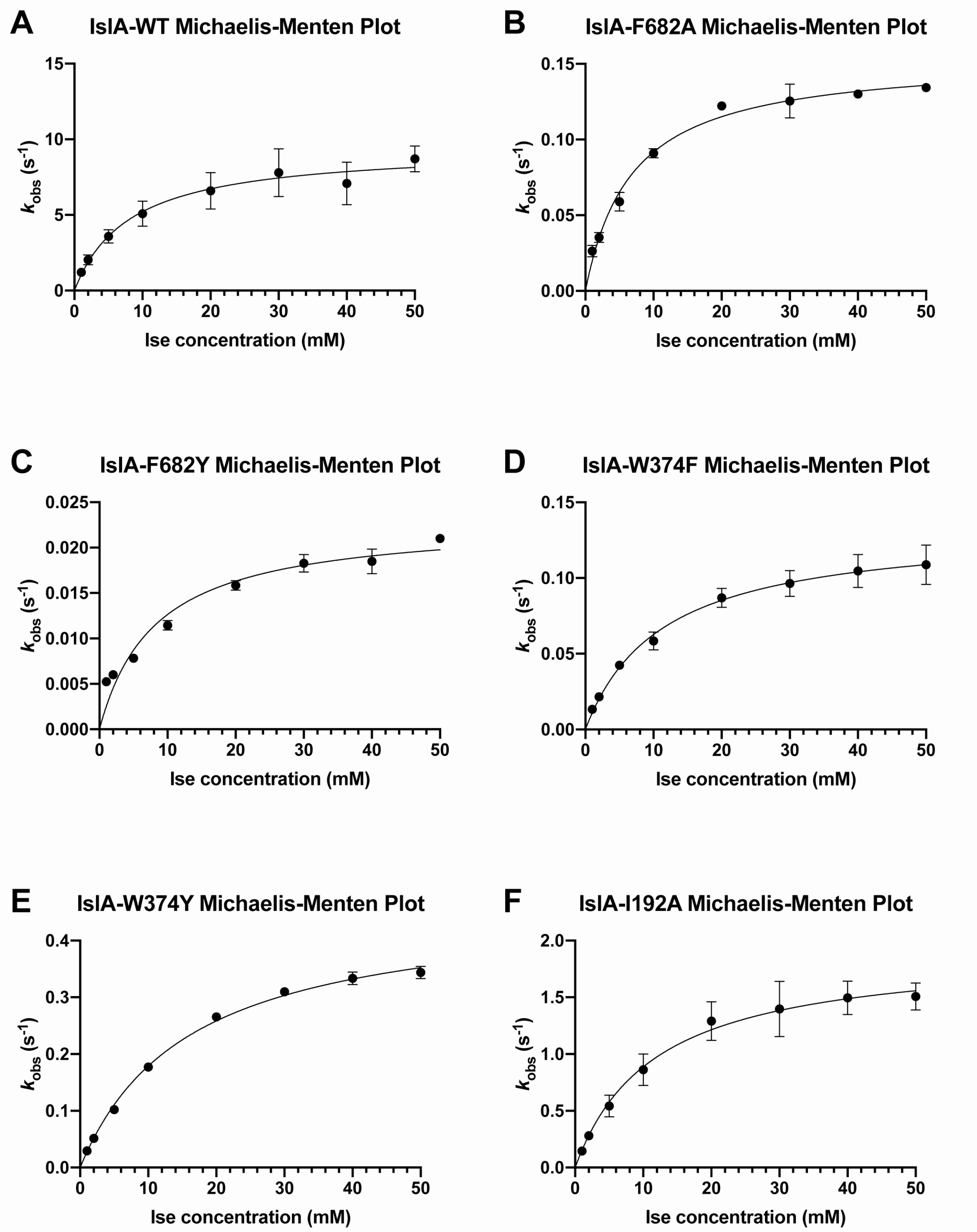

Figure S7, related to Table 2: Michaelis-Menten kinetics for IslA variants. The Michalis-Menten kinetics plots for Ise turnover using the yeast alcohol dehydrogenase coupled assay are shown. (A) IslA-WT has a k_{cat} of 9.5 ± 0.6 s^{-1} and a K_M of 8 ± 2 mM. (B) IslA-F682A has a k_{cat} of 0.154 ± 0.004 s^{-1} and a K_M of 6.8 ± 0.6 mM. (C) IslA-F682Y has a k_{cat} of 0.023 ± 0.001 s^{-1} and a K_M of 8 ± 1 mM. (D) IslA-W374F has a k_{cat} of 0.133 ± 0.006 s^{-1} and a K_M of 11 ± 1 mM. (E) IslA-W374Y has a k_{cat} of 0.465 ± 0.009 s^{-1} and a K_M of 16.0 ± 0.9 mM. (F) IslA-I192A has a k_{cat} of 1.9 ± 0.1 s^{-1} and a K_M of 12 ± 2 mM. Data points indicate mean ± standard deviation of three replicates.

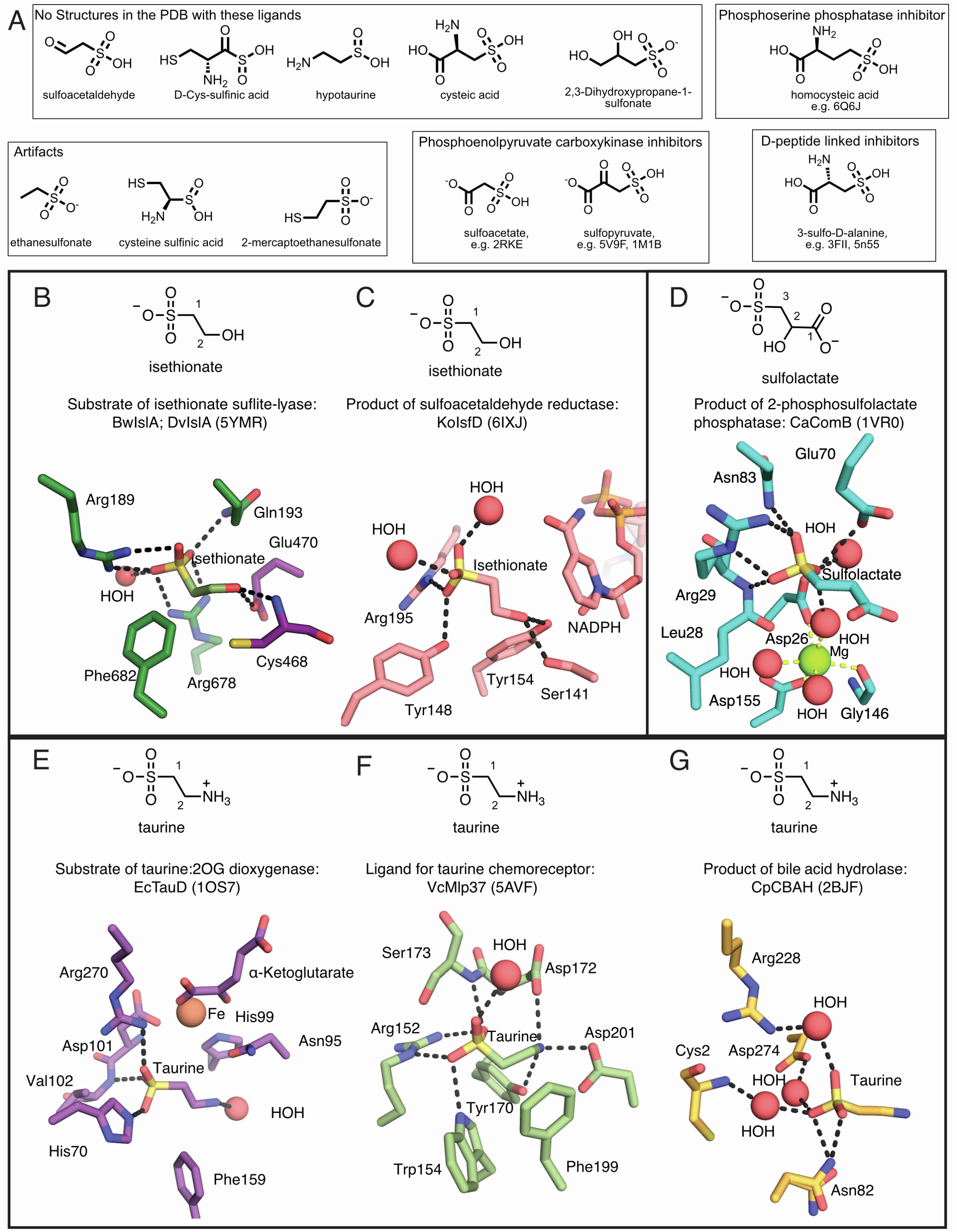

Fig. S8, related to Figure 6: Sulfonates. (A) Sulfonate molecules that are either absent in the PDB or bound in a nonphysiological mode and (B-G) physiological sulfonate binding modes of isethionate, sulfolactate, and taurine as either substrates or products.

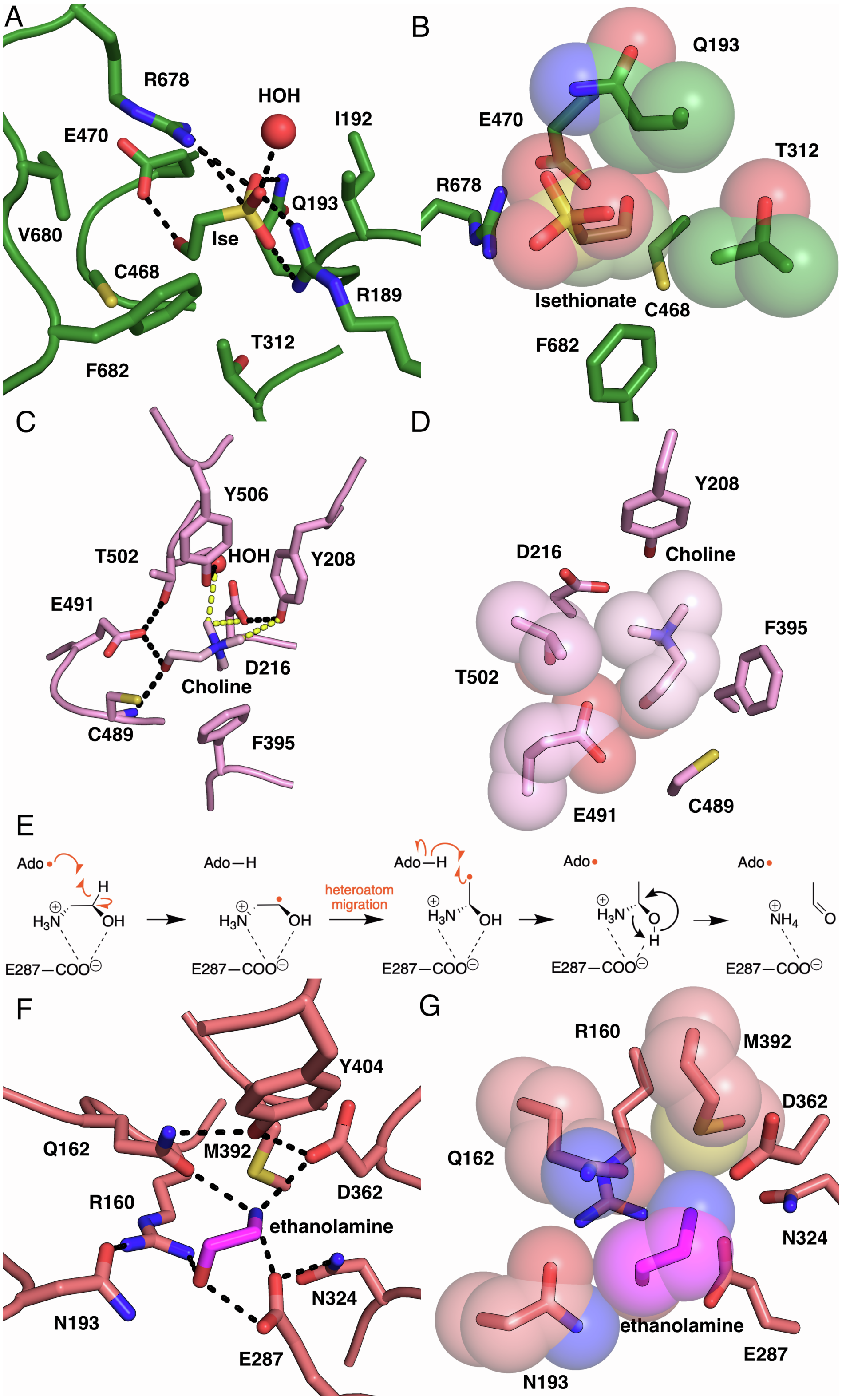

Fig. S9, related to Figure 6: The IslA and CutC active sites appear primed for elimination chemistry, in contrast to the proposed migration chemistry of EAL. Hydrogen bond interactions and CH—O bond interactions are show in black and yellow dashes, respectively. (A) The IslA active site with Ise and nearby residues shown as sticks. (B) Another view of IslA with van der Waals spheres shown for Q193, T312, and Ise. (C) The CutC active site with choline and nearby residues shown as sticks. (D) Another view of CutC with van der Waals spheres shown for T502, E491, and choline. (E) Simplified reaction scheme for EAL (Mori et al., 2014; Toraya, 2003). (F) The EAL active site with ethanolamine and nearby residues shown as sticks (PDB ID 3ABO) (Shibata et al., 2010). (G) Another view of EAL with van der Waals spheres shown for M392, Q162, N193, and ethanolamine.

Table S1, related to Figure 6: Thermodynamic corrections to the electronic energy (E_{el}) in kcal/mol obtained with B3LYP/6-31G*-optimized geometries of acetate, CH_3S•, isethionate, isethionate-acetate complex, and the transitions states (TSes) corresponding to hydrogen atom transfer from isethionate to CH_3S•, both in the presence and absence of acetate. Zero point energy (ZPE) (column 2), ZPE and thermal energy (column 3), entropy multiplied by temperature (TS, where T=298.15 K; column 4), and the energy that is to be added to E_{el} to transform it into G^0 ($G-E_{el}$; column 5) in kcal/mol are shown. Inner energy, $U=ZPE + thermal\ energy + E_{el}$. $G-E_{el}=ZPE + thermal\ energy + k_BT - TS$, where k_B is the Boltzmann constant and T=298.15K.

**System**	**ZPE (kcal/mol)**	**ZPE + thermal energy ($U-E_{el}$) (kcal/mol)**	**Entropy (TS) (kcal/mol)**	**$G-E_{el}$ ($H-TS-E_{el}$ = $U+k_BT-TS-E_{el}$ = ZPE + thermal + k_BT - TS) (kcal/mol)**
acetate	30.32	33.07	20.13	13.53
CH_3S•	23.02	24.94	17.73	7.80
isethionate	52.66	57.42	25.57	32.44
isethionate-acetate	84.56	92.84	34.21	59.22
isethionate-CH_3S (TS)	72.61	79.91	32.23	48.27
isethionate-CH_3S-acetate (TS)	104.78	115.48	40.07	76.00

Table S2, related to Figure 6: Relative gas-phase DLPNO-CCSD(T)/CBS electronic energies (column 2), solvent corrected DLPNO-CCSD(T)/CBS energies with solvent correction energies obtained at the MP2/CBS level of theory for dielectric values of 10 (column 3) and 78.39 (column 4), Gibbs free energies at T=298.15 K and 1 atm pressure for dielectric values of 10 (column 5) and 78.39 (column 6) are shown. All energies reported here are in units of kcal/mol. The two-point extrapolation formula based on the aug-cc-pVDZ and aug-cc-pVTZ energies is used to extrapolate to the complete basis set limit (Helgaker et al., 1997; Neese and Valeev, 2011; Zhong et al., 2008) for DLPNO-CCSD(T). DLPNO-CCSD(T)/CBS energies were computed using tight PNO thresholds, which refer to the default thresholds of TCutPairs = 10^{-5}, TCutPNO = 1.00 x 10^{-7}, and TCutMKN = 10^{-3}.

**System**	**DLPNO-CCSD(T)/CBS (kcal/mol)**	**DLPNO-CCSD(T)/CBS + solv. corr. with ε=10 (kcal/mol)**	**DLPNO-CCSD(T)/CBS + solv. corr. with ε=78.39 (kcal/mol))**	**DLPNO-CCSD(T)/CBS + solv. corr. with ε=10 + G - E_{el} (kcal/mol)**	**DLPNO-CCSD(T)/CBS + solv. corr. with ε=78.39 + G - E_{el} (kcal/mol**
acetate	-143261.94	-143321.66	-143330.71	-143308.13	-143317.18
CH_3S•	-274585.59	-274587.59	-274587.93	-274579.79	-274580.13
isethionate	-487969.01	-488026.40	-488035.18	-487993.96	-488002.74
isethionate-acetate	-631203.22	-631350.92	-631372.76	-631291.70	-631313.54
isethionate-CH_3S (TS)	-762542.57	-762599.13	-762608.08	-762550.86	-762559.81
isethionate-CH_3S-acetate (TS)	-905786.31	-905928.36	-905949.56	-905852.36	-905873.56

Table S3, related to Figure 6: Activation energies of TSes corresponding to hydrogen atom transfer from isethionate to $CH_3S\bullet$ in the presence (row 3) and absence (row 2) of acetate. Activation energies incorporating MP2/CBS solvent corrections to the gas-phase DLPNO-CCSD(T)/CBS electronic energies for dielectric values of 10 (column 2) and 78.39 (column 3) and activation energies obtained from Gibbs free energies of systems for dielectric values of 10 (column 4) and 78.39 (column 5) are shown. All the reported energies are in kcal/mol.

**System**	**Activation energy: DLPNO-CCSD(T)/CBS + solv. corr. with ε=10 (kcal/mol)**	**Activation energy: DLPNO-CCSD(T)/CBS + solv. corr. with ε=78.39 (kcal/mol))**	**Activation energy: DLPNO-CCSD(T)/CBS + solv. corr. with ε=10 + G - E_{el} (kcal/mol)**	**Activation energy: DLPNO-CCSD(T)/CBS + solv. corr. with ε=78.39 + G - E_{el} (kcal/mol**
isethionate-CH_3S (TS)	14.86	15.04	22.89	23.07
isethionate-CH_3S-acetate (TS)	10.15	11.14	19.13	20.12

Table S4, related to Table 2: Oligonucleotides used for cloning.

Oligonucleotide	Target	Sequence (5′ to 3′)
Bwad_IslA_R189E_F	IslA-R189E	gcacccactggatggaggactcgaaagaggaggtttcgttg
Bwad_IslA_R189E_R	IslA-R189E	caacgaaacctcctctttcgagtcctccatccagtgggtgc
Bwad_IslA_I192A_F	IslA-I192A	gtcatgcacccactgggcggaggaacggaaagag
Bwad_IslA_I192A_R	IslA-I192A	ctctttccgttcctccgcccagtgggtgcatgac
Bwad_IslA_Q193A_F	IslA-Q193A	cctccatcgcgtgggtgcatg
Bwad_IslA_Q193A_R	IslA-Q193A	catgcacccacgcgatggagg
Bwad_IslA_W374F_F	IslA-W374F	ccaatggtgacggcttcgaagtgagcgtaaccttcg
Bwad_IslA_W374F_R	IslA-W374F	cgaaggttacgctcacttcgaagccgtcaccattgg
Bwad_IslA_W374Y_F	IslA-W374Y	ccaatggtgacggcttcatagtgagcgtaaccttcg
Bwad_IslA_W374Y_F	IslA-W374Y	cgaaggttacgctcactatgaagccgtcaccattgg
Bwad_IslA_C468S_F	IslA-C468S	gtgtccggcagcatcgaaatc
Bwad_IslA_C468S_R	IslA-C468S	gatttcgatgctgccggacac
Bwad_IslA_E470Q_F	IslA-E470Q	ggctgcatccaaatccgtatg
Bwad_IslA_E470Q_R	IslA-E470Q	catacggatttggatgcagcc
Bwad_IslA_R678E_F	IslA-R678E	gaggtgaagggcacgtactcggcgtcgttgttgatgc
Bwad_IslA_R678E_R	IslA-R678E	gcatcaacaacgacgccgagtacgtgcccttcacctc
Bwad_IslA_F682A_F	IslA-F682A	acgtgagaggtggcgggcacgtaacg
Bwad_IslA_F682A_R	IslA-F682A	cgttacgtgcccgccacctctcacgt
Bwad_IslA_F682Y_F	IslA-F682Y	gcacgtgagaggtatagggcacgtaacgggc
Bwad_IslA_F682Y_R	IslA-F682Y	gcccgttacgtgccctatacctctcacgtgc
Bwad_IslA_G805A_F	IslA-G805A	cgcatcgccgcgtacagcgcc
Bwad_IslA_G805A_R	IslA-G805A	ggcgctgtacgcggcgatgcg
Bwad_IslA_pET28a_F	Overlap Extension PCR	gcagcggcctggtgccgcgcggcagccatatgactc aggtagctgaaatcaaatc
Bwad_IslA_pET28a_R	Overlap Extension PCR	ggatctcagtggtggtggtggtggtgctcgagttac atctggtcgtggccggtacg
